# MATLAB simulation based study on poliovirus sensing through one-dimensional photonic crystal with defect

**DOI:** 10.1038/s41598-023-35595-6

**Published:** 2023-06-09

**Authors:** Arafa H. Aly, B. A. Mohamed, S. K. Awasthi, Suhad Ali Osman Abdallah, A. F. Amin

**Affiliations:** 1 TH-PPM Group, Physics Department, Faculty of Sciences, Beni-Suef University, Beni Suef, 62514 Egypt; 2 Department of Physics and Material Science and Engineering, Jaypee Institute of Information Technology, Noida, 201304 India; 3 Applied College, Khamis Mushait, King Khalid University, Abha, 62529 Saudi Arabia; 4 Faculty of Technology and Education, Beni-Suef University, Beni Suef, 62521 Egypt

**Keywords:** Materials science, Optics and photonics, Physics

## Abstract

The present work, theoretically examined the poliovirus sensor model composed of one-dimensional photonic crystal with defect. The transfer matrix method with the help of MATLAB software has been used to detect poliovirus present in the water sample. The main objective of the present work is to design an efficient sensor by identifying the minute variation in the refractive index of water sample due to change in the poliovirus concentration present in the sample. The alternate layers of aluminum nitride and gallium nitride has been taken to realize Bragg reflector having defect layer of air at center of the Bragg reflector. The effect of change in thickness of defect layer region, period number and incident angle corresponding to transverse electric wave has been examined to optimize the structure which correspond maximum performance of the proposed poliovirus sensing structure. The maximum performance of the structure has been obtained with optimum value of defect layer thickness 1200 nm, period number 10 and incident angle 40°. Under optimum condition maximum sensitivity of 1189.65517 nm/RIU has been obtained when the structure is loaded with waters sample of poliovirus concentration *C* = 0.005 g/ml whereas figure of merit, quality factor, signal to noise ratio, dynamic range, limit of detection and resolution values become 2618.28446 per RIU, 3102.06475, 2.27791, 2090.99500, 1.91E−05 and 0.24656 respectively.

## Introduction

Nowadays optical biosensors based on photonics and plasmonics technologies are being extensively used as a most popular tool for sensing and detection of analytes belong to the field of biomedical applications, food safety, chemical engineering and environmental monitoring. The plasmonics biosensors work on the principle of surface plasma resonance (SPR) either by utilizing angle interrogation or wavelength interrogation. Angle interrogation is preferred over wavelength integration because it provides better results like larger signal-to-noise ratio. The interaction of immobile ligands with analytes on the surface of sensing medium due to change refractive index of analyte results significant change in the resonance angle of plasmonics biosensors based on SPR phenomenon. The resent review article of Arun et al. highlights the advances of biosensors based on SPR technologies for which includes SPR based imagining and magneto-optical sensing. This article also highlights the features of some other important optical biosensors which work on the fiber optic phenomenon like evanescent wave, fluorescence evanescent wave and polymerase colorimetric chain reaction. Some other optical biosensors based on interferometry, ellipsometry, enhanced surface Raman scattering phenomenon are also been discussed^1^. Some of the research fellows have investigated the novel way of designing of optical biosensors by utilizing the phenomenon of long-range SPR^2,3^. For example research work of Arun et al. gives an excellent idea of designing a long-range SPR sensor with improved sensing capabilities employing 2D material layer of platinum diselenide (PtSe2)^3^. Apart from the SPR technology based excellent biosensing devices some of the research groups are giving their efforts to design and develop biosensors based on one-dimensional (1d) photonic crystals (pcs) due their feasibility of fabrication, simple architecture, user friendly handling and cost effectiveness.

Photonic structures are the smart composites which could tremendously control the propagation of light due to appearance of novel phenomenon of photonic bandgaps (PbGs). All the frequencies of light incident on such structures are significantly attenuated if they fall inside this gap^4–7^. The photon localization and existence of PbG are amongst the peculiar properties associated with the photonic structures^8,9^. The ease of designing and fabrication makes 1d photonic structures as a most suitable and promising candidates over two-dimensional (2d) and three-dimensional (3d) structures^10^. Any deformation in the periodicity of such structures may result appearance of resonant tunneling mode inside PbGs which can be easily relocated inside PbG either by changing the refractive index or by changing the thickness of defect layer region theoretically as well as experimentally^11,12^. Thus the deformation in the periodicity of photonic designs may enhance the novelty in comparison to that of perfect ones which have attracted the optical and physical societies for the development of various sensors with remarkable relevance in optical, chemical, physical and biomedical applications^13^. Such aperiodic structures are called as defective photonic crystals (dpcs)^14^. The sensing and identification capabilities of dpcs have opened new gateway for the development of various sensors like biosensors, salinity sensor, chemical sensor, pressure sensor and magnetic sensor which have their usefulness in wide range of biological, chemical, medical and environmental applications^15–19^. This usefulness is due to their excellent set of properties like higher sensitivity, quick response time and large quality factors^20,21^. The most of the applications discussed above are based on the movement of resonant mode inside PbG due to variation in the refractive index of defect layer region in presence of specific external environment like concentration, temperature, magnetic field and pressure^22,23^. In particular, the refractive index sensing may be the cornerstone for identification and detection of various samples depending upon their physical, biological and chemical properties^12,24^.

The present study is based on theoretical examination of the transmission properties of 1d dpc loaded with water sample containing poliovirus with the help of MATLAB software. The objective of this research work is to develop a cost effective alternative sensor composed of 1d dpc for detection poliovirus sensor because it destroy the nerve cells and may become root cause of acute flaccid paralysis (AFP)^25^. The intention of the present work as per our believe is to develop a simple and new method involving low fabrication cost in comparison to the conventional methods like cell culture, intratypic differentiation, genome sequencing, serology for poliovirus diagnostic^26^. The results of these traditional methods have poor stability, lower accuracy, high cost and required specific environmental conditions during investigation process^27^. On the other hand the present work only deals with the change in the properties of resonant mode due to change in the refractive index of poliovirus sample. The cornerstone of the work presented here deals with the refractive index variation of water sample due to change in its poliovirus concentration. Any minute variation in the refractive index of the sample is immediately sensed by relocating the position of resonant peak inside PbG of the sensor. The numerical results of this work are based on transfer matrix method and change in the refractive index of water dependent upon the poliovirus concentration.

The organized of the manuscript is as under: the poliovirus sensor design and structural formulation of the work are briefly discussed in “Structural design and its realization” and “Description of poliovirus solution”. The results and discussions pertaining to the poliovirus sensor are presented in “Results and discussions”. Finally we have discussed the conclusions in “Conclusions”.

## Structural design and its realization

**Figure 1:**
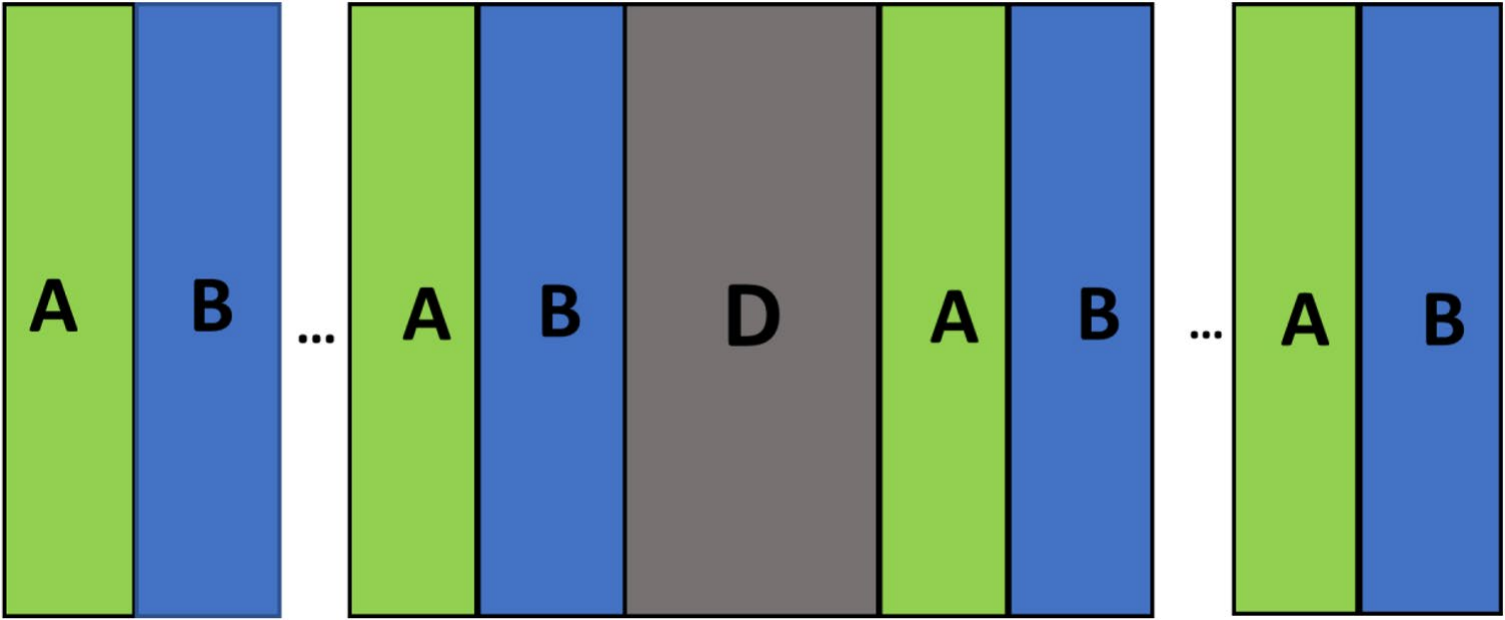
Structural design of proposed 1d defective planar photonic crystal (AB)^N^D(AB)^N^. Here the symbols *A*, *B* and *D* are representing materials Aluminum nitride (ALN), Gallium nitride (GaN) and air cavity respectively with period number N.

The proposed poliovirus sensor has been made by using 1d defective photonic crystal (AB)ND(AB)N as shown in Fig. 1 below.

The letters *A*, *B* and *D* have been used to represent materials Aluminum nitride (ALN), Gallium nitride (GaN) and air cavity of the proposed design respectively^28,29^. The air cavity region *D* of the structure is utilized for investigating various poliovirus samples poured into *D*. The spectral dependence of refractive indices of aluminum and gallium nitride materials in the infrared region of spectrum has been defined with the help of Eqs. (1) and (2) respectively as^28,29^

1 $$n_{A}^{2} - 1 = 2.1399 + \frac{{13786\lambda^{2} }}{{\lambda^{2} - 0.1715^{2} }} + \frac{{3.861\lambda^{2} }}{{\lambda^{2} - 15.03^{2} }}$$

2 $$n_{B}^{2} - 1 = 2.60 + \frac{{1.75\lambda^{2} }}{{\lambda^{2} - 0.256^{2} }} + \frac{{4.1\lambda^{2} }}{{\lambda^{2} - 17.86^{2} }}$$

Here *n*_A_ and *n*_B_ are representing refractive indices of aluminum and gallium nitride materials respectively. The free space wavelength is measured in nanometers. The thickness of layers A, B and D are taken to be *d*_A_ = 100 nm, *d*_B_ = 150 nm and *d*_D_ = 800 nm respectively. The period number N of the structure has been initially selected as 9 to begin with simulation work.

There are several fabrication techniques that can be used for realization of such defective photonic structures. The materials choice is very essential for identifying the most suitable fabrication technique to realize such structures like dip coating, sol–gel method etc. The fabrication of multilayer periodic structures made up of materials like nanocomposite, polymers, and dielectrics can be easily realized by using a process of spin coating. In this process first we apply precursor solution over the flat substrate, the evaporation of the solvent from the substrate is done by initiating the spin process. Next annealing protocol is applied for solicitation of the layer on the substrate. The remaining stacks of layers are being deposited one over the other by repeating above mentioned sequence of various steps involved in the deposition of layers. During the deposition of various layers we also control the thickness of each layer of the stack by either monitoring the concentration of cast solution or by controlling the rotational speed. After fabricating the structure one can use standard elliposmetric spectroscopic methods dependent upon angle for characterization of the fabricated structure.

## Description of poliovirus solution

The refractive index of the contaminated water with poliovirus (*n*_pv_) dependent upon the various poliovirus concentration levels (*C*) measured in g/ml present in the water is defined in Eq. (3) as^30^

3 $$n_{pv} = n_{w} + aC$$

Here *n*_w_ is representing refractive index of pure water (1.33) and *a* is the specific refractive index of water contaminated with poliovirus of specific concentration level 0.174 ml/g. In the present research work the concentration level *C* of the virus has been varied from 0.001 to 0.05 g/ml in steps of 0.001. All the concentration dependent refractive index values of water contaminated with poliovirus has been presented in Table 1 below. These values have been extracted from Eq. (3).

**Table 1:** Tabulated refractive index values of poliovirus dependent upon various concentration levels.

*C* (g/ml)	n_pv_
0	1.33
0.001	1.330174
0.002	1.330348
0.003	1.330522
0.004	1.330696
0.005	1.330870
0.006	1.331044
0.007	1.331218
0.008	1.331392
0.009	1.331566
0.010	1.331740
0.011	1.331914
0.012	1.332088
0.013	1.332262
0.014	1.332436
0.015	1.332610
0.016	1.332784
0.017	1.332958
0.018	1.333132
0.019	1.333306
0.020	1.333480
0.021	1.333654
0.022	1.333828
0.023	1.334002
0.024	1.334176
0.025	1.334350
0.026	1.334524
0.027	1.334698
0.028	1.334872
0.029	1.335046
0.030	1.335220
0.031	1.335394
0.032	1.335568
0.033	1.335742
0.034	1.335916
0.035	1.336090
0.036	1.336264
0.037	1.336438
0.038	1.336612
0.039	1.336786
0.040	1.336960
0.041	1.337134
0.042	1.337308
0.043	1.337482
0.044	1.337656
0.045	1.337830
0.046	1.338004
0.047	1.338178
0.048	1.338352
0.049	1.338526
0.050	1.338700

### Computational model

In the present design we have considered 1d Bragg reflector whose periodicity has been broken by considering a defect layer of air at the centre of Bragg reflector to get modified 1d dpc (AB)^*N*^D(AB)^*N*^ as discussed above in Fig. 1. All the layers of the proposed poliovirus sensing structure are deposited over a glass substrate of refractive index *n*_S_. The surrounded medium around whole structure is air. The plane polarized light whose electric field wave vector is normal to plane of incidence is allowed to enter into the 1d dpc (AB)^*N*^D(AB)^*N*^at an incident angle θ with normal to the senor from air^31^.

The transfer matrix is used here for describing the interaction between the incident light with the proposed structure as^32^

4 $$C=\left(\begin{array}{cc}{C}_{11}& {C}_{12}\\ {C}_{21}& {C}_{22}\end{array}\right)={\left({c}_{A}{c}_{B}\right)}^{N}({c}_{D}){({c}_{A}{c}_{B})}^{N}$$

Here transfer matrix elements are being represented by ${\mathrm{C}}_{11}, {\mathrm{C}}_{12}, {\mathrm{C}}_{21}\mathrm{ and }{\mathrm{C}}_{22}$. The period number is shown by a letter N. The interaction between incident S polarized light with proposed poliovirus sensor is studied by TMM. There are many articles in which details of TMM can be found. The interplay between proposed sensor and S polarized can be defined with the help of following matrix as^33^

5 $${c}_{j}=\left(\begin{array}{cc}{cos\gamma }_{j}& -i/{q}_{j}sin{\gamma }_{j}\\ -i{q}_{j}sin{\gamma }_{j}& -i/{q}_{j}sin{\gamma }_{j}\end{array}\right),$$

The subscript *j* is used to represent layers *A*, *B* and *D* of the structure (*j* $=A, B\mathrm{and} D).$ For S polarized incident light one can define *q*_j_as ${q}_{j}={n}_{j}cos{\gamma }_{j}$. Here phase difference in each layer of the sensor is denoted by ${\gamma }_{j}$ as

6 $${\gamma }_{j}=\frac{2\pi {n}_{j}{d}_{j}cos{\theta }_{j}}{\lambda }$$

Here the index of refraction, thickness and ray angle inside *j* layer of the poliovirus sensor are represented by *n*_j_, *d*_j_ and *θ*_*j*_ respectively.

One can easily obtained the coefficient of transmission *t* of proposed poliovirus sensor by using the relation as under^33^

7 $$t = \frac{{2q_{0} }}{{(C_{11} + C_{12} q_{S} )q_{0} + (C_{21} + C_{22} q_{S} )}}$$

Here notations $q_{0} = n_{0} \cos \theta$ and $q_{S} = n_{S} \cos \theta_{S}$ are being used to represent phase differences associated with light wave in incident and exit media respectively for TE polarized wave.

The transmittance (*T*) of our poliovirus sensor can be obtained by^33^

8 $$T = \frac{{q_{S} }}{{q_{0} }}\left| t \right|^{2}$$

## Results and discussions

**Figure 2:**
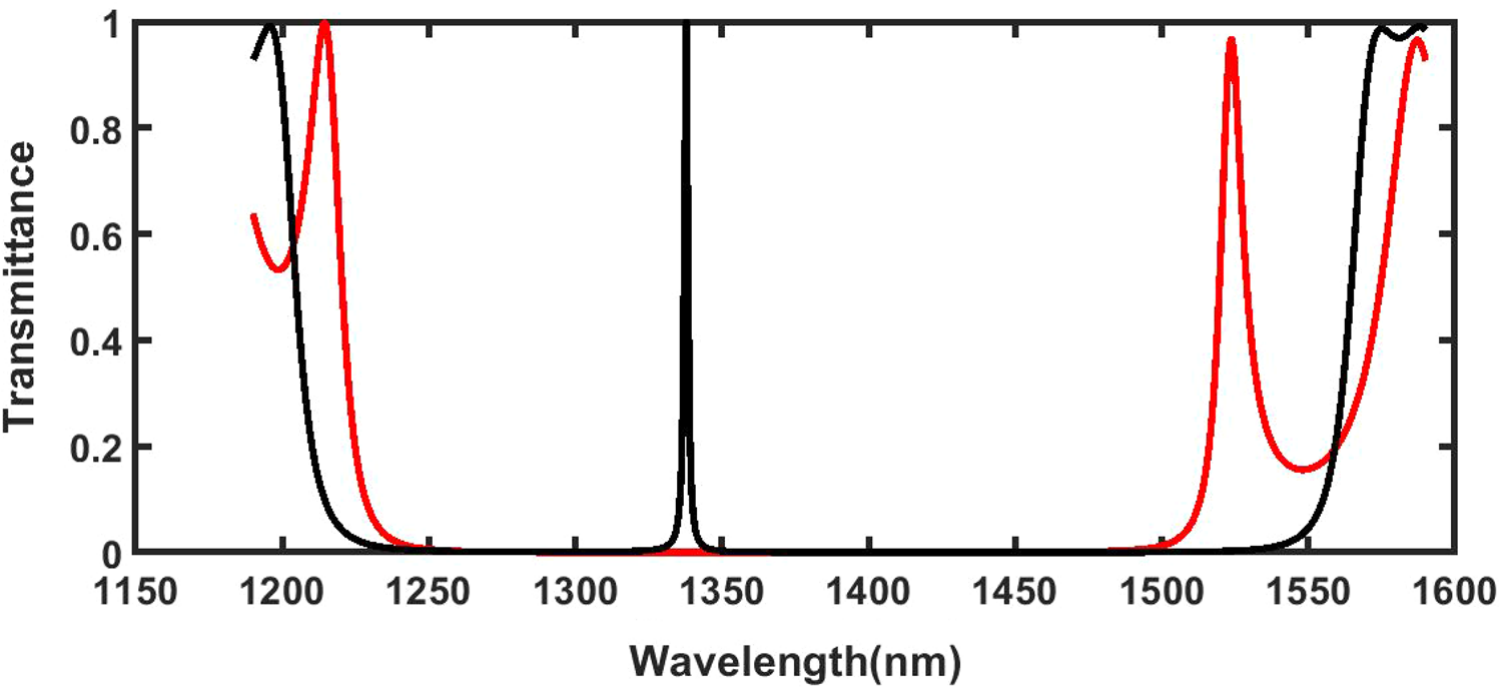
Transmittance spectra of 1d photonic structure (**a**) (AB)^18^ without cavity layer D and (**b**) (AB)^9^D(AB)^9^ with cavity layer Dof thickness *dd* = 800 nm under normal incidence. The structure (AB)^9^D(AB)^9^ is loaded with pure water sample of refractive index 1.330.

With the help of theoretical formulation discussed above we have developed MATLAB code for evaluation of transfer matrix of the structure (AB)^9^D(AB)^9^ which in turn provides the transmission characteristics and other relevant plots pertaining to the proposed work. First the simulations has been initiated by pouring a water sample of refractive index 1.33 into the air cavity reason D of the structure and allow a light to enter into the structure normally. The entire simulation work has been carried out in the infrared region of electromagnetic spectrum ranging from 1190 to 1590 nm. Figure 2 below shows the transmittance of the structures (AB)^18^ without cavity layer D and (AB)^9^D(AB)^9^ with cavity layer D loaded with water sample of refractive index 1.33. Figure 2 contains two transmittance plots corresponding to structures without and with cavity layer D loaded with water sample in red and black colour solid lines respectively. The transmittance plot of the structure (AB)^18^ shows PBG located between 1240 and 1485 nm. The introduction of cavity layer D into the middle of one dimensional multilayer periodic structure (AB)^18^ is responsible of the creation of 1d defective photonic crystal structure (AB)^9^D(AB)^9^ as shown in Fig. 1. This modification ensures the appearance of discontinuous electromagnetic radiations of specific wavelength knows as resonant mode or defect mode inside PBG of 1d dpc. The presence of resonant mode inside PBG of 1d dpc is due to the break in periodicity of the structure. The transmittance plot shown in black solid line in Fig. 2 is representing the PbG of 1d dpc (AB)^9^D(AB)^9^ in addition to resonant mode of unit transmittance located at wavelength 1337.7418 nm inside PbG. In this research work we have explored the biosensing capabilities of the defect mode inside PbG associated with 1d defective pc for the sensing and detection of poliovirus present in the water sample.

### Reflectance spectra of 1d dPhC (AB)^9^D(AB)^9^ loaded with water samples of low concentration

In all the reflectance calculations of the 1d DPC (AB)^9^D(AB)^9^ the thickness of layers A, B and D are initially taken as *d*_A_ = 100 nm, *d*_B_ = 150 nm and *d*_D_ = 800 nm respectively.

**Figure 3:**
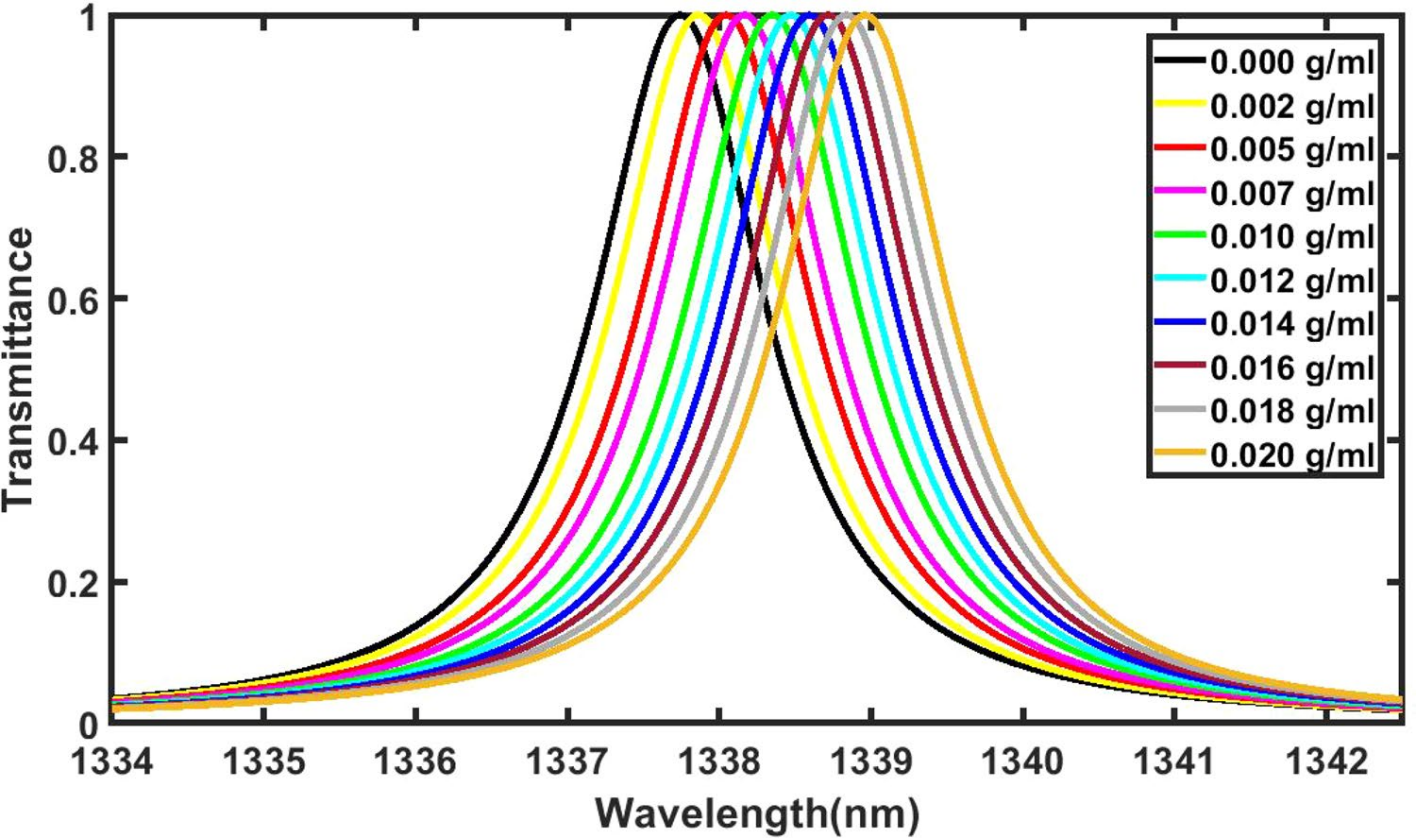
Reflectance spectra of 1d DPC (AB)^9^D(AB)^9^ loaded with water samples containing poliovirus of low concentration level as 0.002 g/ml, 0.005 g/ml, 0.007 g/ml, 0.010 g/ml, 0.012 g/ml, 0.014 g/ml, 0.016 g/ml, 0.018 g/ml and 0.020 g/ml with respect to pure water sample of concentration 0 g/ml.

Figure 3 below shows the reflectance of 1d DPC (AB)^9^D(AB)^9^ loaded with water samples containing poliovirus of low concentration level as 0.002 g/ml, 0.005 g/ml, 0.007 g/ml, 0.010 g/ml, 0.012 g/ml, 0.014 g/ml, 0.016 g/ml, 0.018 g/ml and 0.020 g/ml with respect to pure water sample of concentration 0 g/ml. In this study normal incidence has been taken and period number is fixed to 9. It is evident from Fig. 3 that as concentration of poliovirus present in the water sample increases the defect mode associated with each water sample shifts towards higher wavelength side. This variation does not have any significant impact on the full width half maximum of each defect mode. It is almost stagnant.

**Figure 4:**
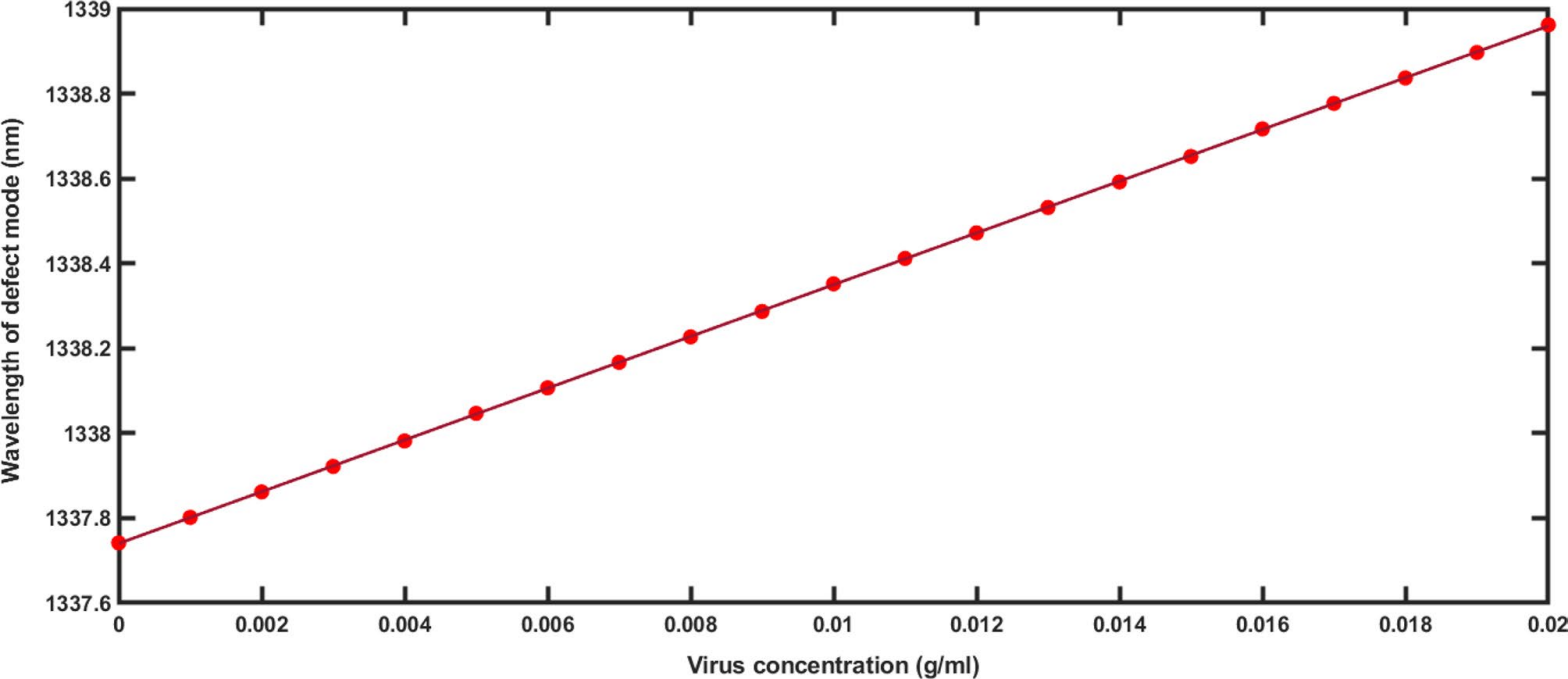
Linear curve fitting applied between poliovirus concentration and the central wavelength of defect mode positions. The red solid balls are representing the observed data extracted from Fig. 3 and red solid line is representing linear curve fitting data.

The change in the position of defect mode inside PBG of 1d DPC (AB)^9^D(AB)^9^ loaded with water samples containing poliovirus of low concentration levels as 0 g/ml, 0.002 g/ml, 0.005 g/ml, 0.007 g/ml, 0.010 g/ml, 0.012 g/ml, 0.014 g/ml, 0.016 g/ml, 0.018 g/ml and 0.020 g/ml are being shown in Fig. 4 below. The change in refractive index of water samples leads to the shifting of defect mode position towards higher wavelength side. Figure 4 shows the linear curve fitting between the positions of poliovirus concentration and the central wavelength of defect mode shown in solid red balls. This linear curve fitting provides a solid red line of slope 60.947 nm/g/ml representing an average sensitivity of our poliovirus sensor as per the following equation

9 $$\lambda_{D} = 60.947C + 1337.7,\,\,(R^{2} = 0.99998)$$

The sensitivity (*S*) and quality factor (*QF*) of any biosensor are amongst the most important parameters which are used for estimating the performance of biosensor. The numeric values of S and QF are calculated with the following equations as^34,35^

10 $$S = \frac{{\Delta \lambda_{D} }}{\Delta n}$$

and

11 $$QF = \frac{{\lambda_{D} }}{FWHM}$$

Here $\Delta \lambda_{D}$ is the change in defect mode position due to change in the refractive index of analytes ($\Delta n$). The symbols $\lambda_{D}$ FWHM are representing central wavelength of defect mode and its full width half maximum respectively.

Further we have evaluated the performance of proposed 1d DPC (AB)^9^D(AB)^9^ loaded with poliovirus solution of different concentration which varies from 0 to 0.05 in steps of 0.001. The purpose we have fulfilled by examining the sensitivity and quality factor of the proposed design loaded with all poliovirus solution as per the data given in Table 2. The data of Table 2 reflects that as the concentration of the poliovirus present in the water sample changes from 0 to 0.050 g/ml in steps of 0.001 the sensitivity of the sensor varies between minimum of 344.8275862 nm/RIU to maximum of 351.2901712 nm/RIU. The variation of quality factor varies between 977.4712949 and 998.7525992 when the concentration of the sample loaded into the structure varies from 0 to 0.05 g/ml.

**Table 2:** Performance evaluation of 1d dpc (AB)^9^D(AB)^9^ loaded with poliovirus solution of different concentration in terms of sensitivity and quality factor.

*C* (g/ml)	*n*_pv_	*λ*_D_ (nm)	*S* (nm/RIU)	*QF*
0	1.33	1337.7418	…	977.4712949
0.001	1.330174	1337.8018	344.8275862	977.9049194
0.002	1.330348	1337.8618	344.8275862	978.3338818
0.003	1.330522	1337.9218	344.8275862	978.7714164
0.004	1.330696	1337.9818	344.8275862	979.1985539
0.005	1.330870	1338.0469	350.6896552	979.5508719
0.006	1.331044	1338.1069	349.7126437	980.0791032
0.007	1.331218	1338.1669	349.0147783	980.5036728
0.008	1.331392	1338.2269	348.4913793	980.9321674
0.009	1.331566	1338.2869	348.0842912	981.3645963
0.010	1.331740	1338.3519	350.6321839	981.7941936
0.011	1.331914	1338.4119	350.1044932	982.2125271
0.012	1.332088	1338.4719	349.6647510	982.6409185
0.013	1.332262	1338.5319	349.2926614	983.0999705
0.014	1.332436	1338.5919	348.9737274	983.5138829
0.015	1.332610	1338.6519	348.6973180	983.9389549
0.016	1.332784	1338.7169	350.2514368	984.3651377
0.017	1.332958	1338.7769	349.9323867	984.8147739
0.018	1.333132	1338.8369	349.6487867	985.2430292
0.019	1.333306	1338.8969	349.3950393	985.6643624
0.020	1.333480	1338.9619	350.6034483	986.0875220
0.021	1.333654	1339.0219	350.3284072	986.5189491
0.022	1.333828	1339.0819	350.0783699	986.9485329
0.023	1.334002	1339.1419	349.8500750	987.3493327
0.024	1.334176	1339.2019	349.6408046	987.8050651
0.025	1.334350	1339.2669	350.5977011	988.2284057
0.026	1.334524	1339.3269	350.3757737	988.6520263
0.027	1.334698	1339.3869	350.1702852	989.0759722
0.028	1.334872	1339.4469	349.9794745	989.5038987
0.029	1.335046	1339.5119	350.7927071	989.9204893
0.030	1.335220	1339.5719	350.5938697	990.3644449
0.031	1.335394	1339.6319	350.4078606	990.7750508
0.032	1.335568	1339.6919	350.2334770	991.1932938
0.033	1.335742	1339.7519	350.0696621	991.6081830
0.034	1.335916	1339.8169	350.7606491	992.0381024
0.035	1.336090	1339.8769	350.5911330	992.4668549
0.036	1.336264	1339.9369	350.4310345	992.8841679
0.037	1.336438	1339.9969	350.2795899	993.2946526
0.038	1.336612	1340.0619	350.8923170	993.7352337
0.039	1.336786	1340.1219	350.7368111	994.1483372
0.040	1.336960	1340.1819	350.5890805	994.5617472
0.041	1.337134	1340.2469	351.1494253	994.9784374
0.042	1.337308	1340.3069	350.9989053	995.3954194
0.043	1.337482	1340.3669	350.8553863	995.8334293
0.044	1.337656	1340.4269	350.7183908	996.2295801
0.045	1.337830	1340.4919	351.2260536	996.6556629
0.046	1.338004	1340.5519	351.0869565	997.0859145
0.047	1.338178	1340.6119	350.9537784	997.4939359
0.048	1.338352	1340.6719	350.8261494	997.8994324
0.049	1.338526	1340.7369	351.2901712	998.3148920
0.050	1.338700	1340.7969	351.1609195	998.7525992

**Figure 5:**
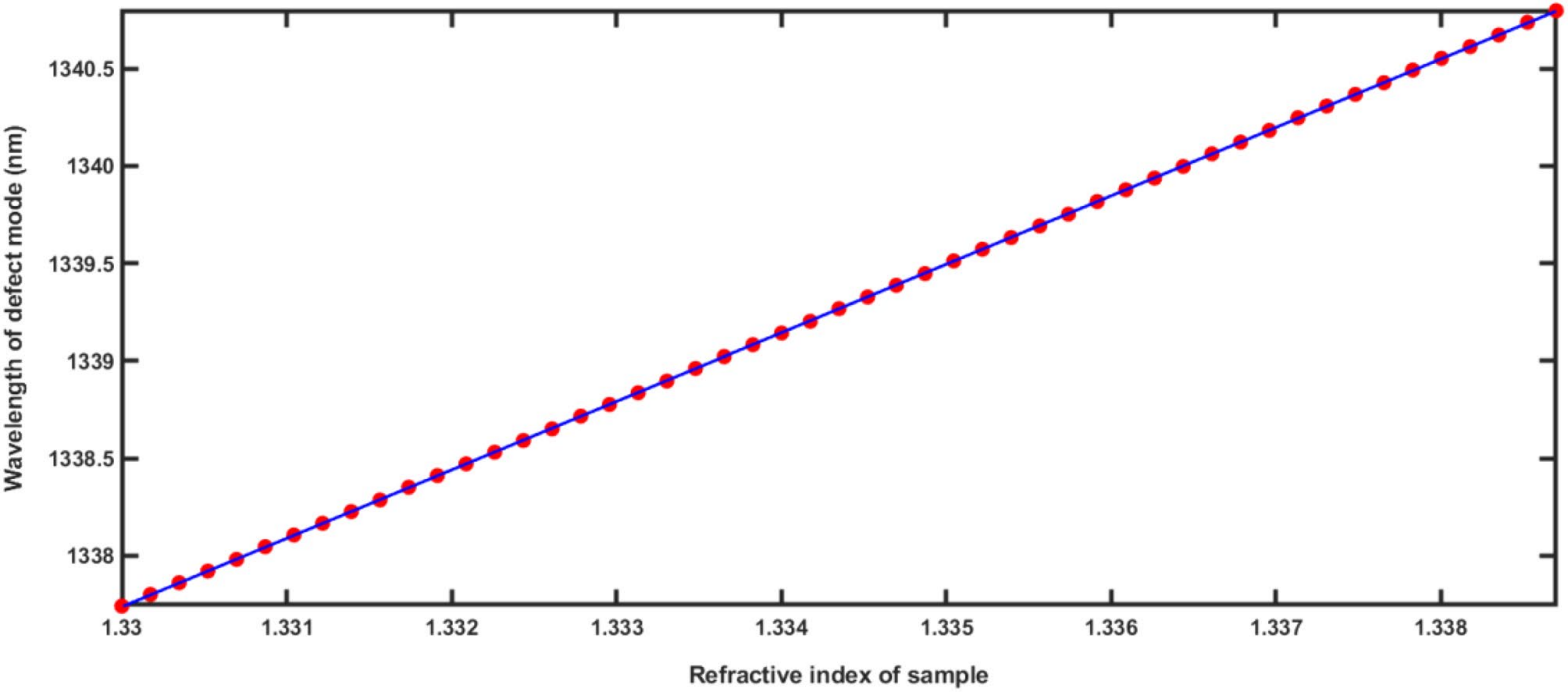
Linear curve fitting applied between the refractive index of poliovirus samples and the central wavelength of defect mode positions. The red solid balls are representing the observed data as described in Table 2 and red solid line is representing linear curve fitting data.

Figure 5 presents the linear curve fitting applied between the refractive index of various poliovirus samples of concentrations 0–0.050 g/ml loaded into the structure and their respective defect mode positions. The slope of line plotted by applying linear curve fitting over the refractive index of poliovirus samples and defect mode positions data as shown in Table 2 refers the average sensitivity of 351.28 nm/RIU of our sensing structure according to the following relation as

12 $$\lambda_{D} = 351.28n_{pv} + 870.536,\,\,\,\,\,(R^{2} = 0.999996)$$

### Effect of concentration of poliovirus present in the water sample on the sensitivity

**Figure 6:**
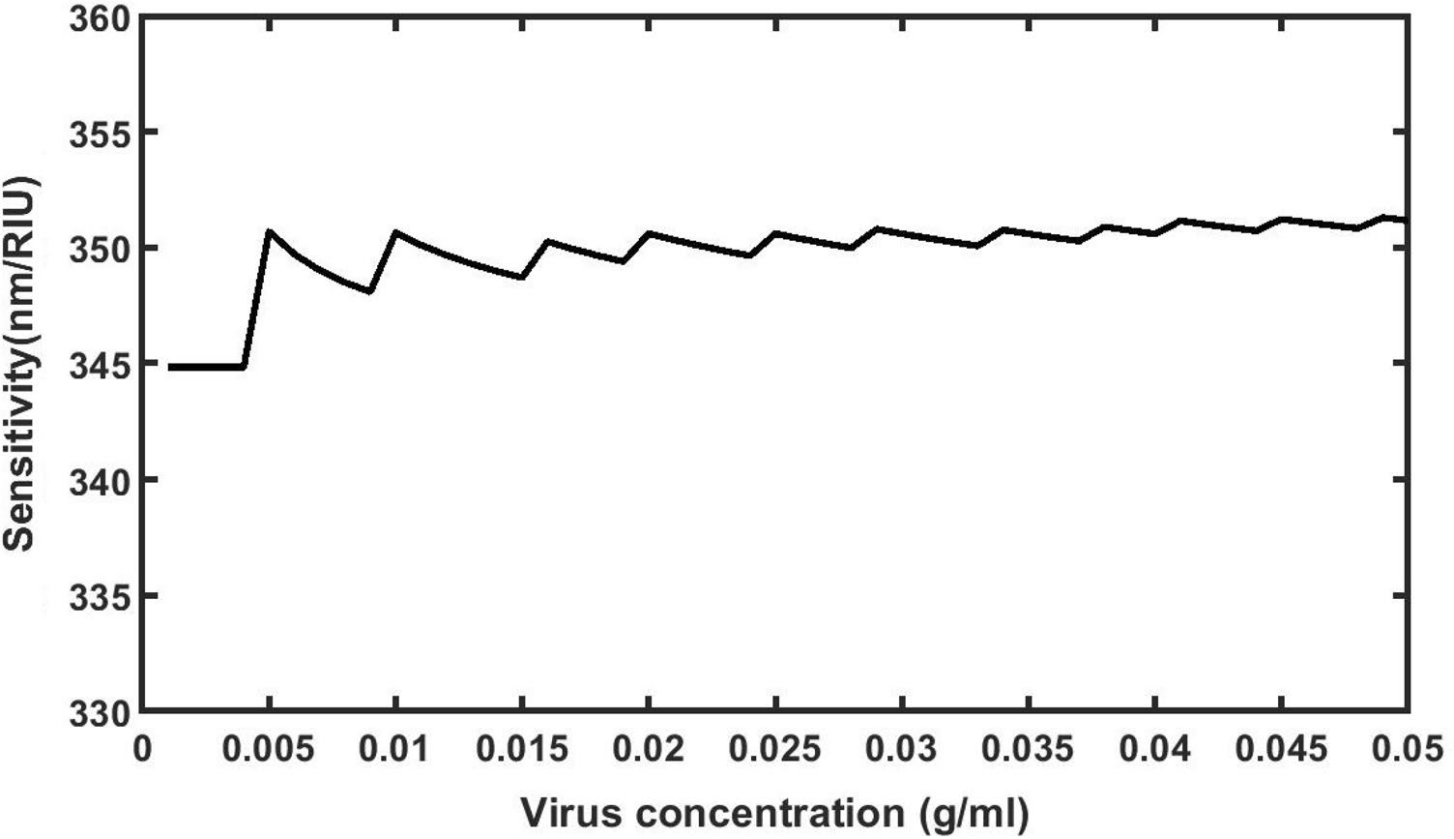
Poliovirus concentration dependent sensitivity of the proposed 1d DPC (AB)^9^D(AB)^9^ loaded with sample of different concentration levels which varies from 0 to 0.05 in steps of 0.001.

According to Eq. (3), the refractive index of water sample contaminated with poliovirus can be directly altered by increasing the concentration of poliovirus present in the sample^30^. The impact of changing the concentration from 0 to 0.05 g/ml on the sensitivity of the proposed sensor composed of 1d DPC will be studied here. Figure 6 shows the sensitivity variation of the proposed structure at different values of concentration level *C* form 0 to 0.05 g/ml as evident from Table 2.

Initially with increase of *C* from 0 to 0.005 g/ml, the sensitivity of proposed sensor increases from 344.8275862 to 350.6896552 nm/RIU due to movement of defect mode associated with the sample towards higher wavelength side as evident from Table 2. Further increase in the value of *C* results some modulated variations of sensitivity which are limited between 348.0842912 and 351.2901712 nm/RIU. These sensitivity variations of our design are very close to average sensitivity value of 351.28 nm/RIU obtained from liner curve fitting as discussed above in Eq. (9).

### Efficiency analysis of the proposed sensor

We have further analyzed the efficiency of proposed sensor composed of 1d DPC (AB)^9^D(AB)^9^ with the help of signal-to-noise ratio, limit of detection and resolution limit. The major factor which influences the performance of any sensing structure is the ratio between signal-to-noise and abbreviated as SNR. In designing of any sensor it is always curtail to have smaller full width half maximum (FWHM) and larger SNR values. The following relation can be used to calculate SNR values of the proposed sensor with the help of Table 2 as^36^

13 $$SNR = \frac{{\Delta \lambda_{D} }}{\Delta n}$$

The finest measurable refractive index variation of the sample is known in terms of limit of detection (LoD). The smallest LoD value of our poliovirus sensor with *d*_D_ = 2000 nm is recorded as 7.16E−05. The LoD values of our structure can be obtained from the following relation by using the data of Table 2 as^37^

14 $$LoD = \frac{{\lambda_{D} }}{20 \times S \times QF}$$

The ability of the poliovirus sensing structure to notice the smallest movement of defect mode inside photonic band gap due to change in the refractive index of the poliovirus sample is defined as resolution limit (RL) and can be calculated by using the following relation as^38^

15 $$RL = \frac{2FWHM}{{3(SNR)^{0.25} }}$$

The change in sensitivity parameter of the poliovirus sensor with respect to change in the full width at half maximum (FWHM) of sensor due to change in analyte is defined as another characteristic parameter called as the figure of merit (FOM). It is defined as^38^

16 $$FoM(RIU^{ - 1} ) = \frac{S(nm/RIU)}{{FWHM(nm)}}$$

The ratio of central wavelength of defect mode with square root of FWHM is measure of dynamic range (DR) of present poliovirus sensor. It is unit less parameter which is defined as^38^

17 $$DR = \frac{{\lambda_{D} (nm)}}{{\sqrt {FWHM(nm)} }}$$

### Optimization of the thickness of defect layer

In addition to sensitivity and quality factor as defined Eqs. (10) and (11), we have included more parameters as defined in Eqs. (13)–(17) for the efficiency analysis of proposed 1d DPC (AB)^9^D(AB)^9^ loaded with sample of concentration *C* = 0.005 (*n*_pv_ = 1.33087). These parameters have been taken as a key factor for optimizing the performance of the structure under the effect of change in the thickness of defect layer, change in period number of the structure and change in angle of incidence. In this analysis we have varied the thickness of defect layer region form *d*_D_ = 800 nm to *d*_D_ = 12,000 nm. The effect of change in thickness of defect layer on the performance evaluating parameters of the structure as defined in previous equations have been recorded in Table 3 below.

**Table 3:** The effect of change in thickness of defect layer of 1d DPC (AB)^9^*D*(AB)^9^ loaded with sample of *C* = 0.005 g/ml on performance evaluating parameters of the proposed sensor under normal incidence.

d_D_ (nm)	FWHM (nm)	S (nm/RIU)	FOM (RIU^-1^)	QF	SNR	DR	LOD	RL
800	1.3660	350.6897	256.7312	979.5509	0.2234	1144.8515	1.95E−04	1.3247
820	1.2783	367.8161	287.7362	1055.0313	0.2503	1192.8434	1.74E−04	1.2048
840	1.2202	385.0575	315.5629	1114.2070	0.2745	1230.7948	1.58E−04	1.1238
860	1.1897	408.0460	342.9926	1152.2432	0.2984	1256.7715	1.46E−04	1.0731
880	1.1860	419.5402	353.7528	1165.4742	0.3078	1269.2276	1.41E−04	1.0615
1000	1.8835	465.5172	247.1543	771.2114	0.2150	1058.4181	2.02E−04	1.8440
2000	0.9223	643.6782	697.9117	1534.6043	0.6072	1473.7729	7.16E−05	0.6965
3000	0.6465	712.6437	1102.3871	2158.7158	0.9591	1735.6598	4.54E−05	0.4355
4000	0.5156	770.1149	1493.5451	2682.7095	1.2994	1926.3813	3.35E−05	0.3220
5000	0.4366	804.5977	1842.7411	3149.0838	1.6032	2080.8533	2.71E−05	0.2587
6000	0.3823	827.5862	2164.9234	3581.1878	1.8835	2214.1776	2.31E−05	0.2175
7000	0.3418	844.8276	2471.5608	3991.6604	2.1503	2333.7373	2.02E−05	0.1882
8000	0.3102	856.3218	2760.8163	4387.4423	2.4019	2443.4934	1.81E−05	0.1661
9000	0.2847	879.3103	3088.5347	4769.8283	2.6870	2545.0575	1.62E−05	0.1482
10,000	0.2630	879.3103	3342.8642	5153.5582	2.9083	2643.1323	1.50E−05	0.1343
11,000	0.2448	896.5517	3662.1062	5529.0407	3.1860	2735.7222	1.37E−05	0.1222
12,000	0.2225	948.2759	4261.5154	6312.2083	3.7075	2977.6019	1.17E−05	0.1069

### Effect of change in thickness of defect layer on *S *and *FWHM*

**Figure 7:**
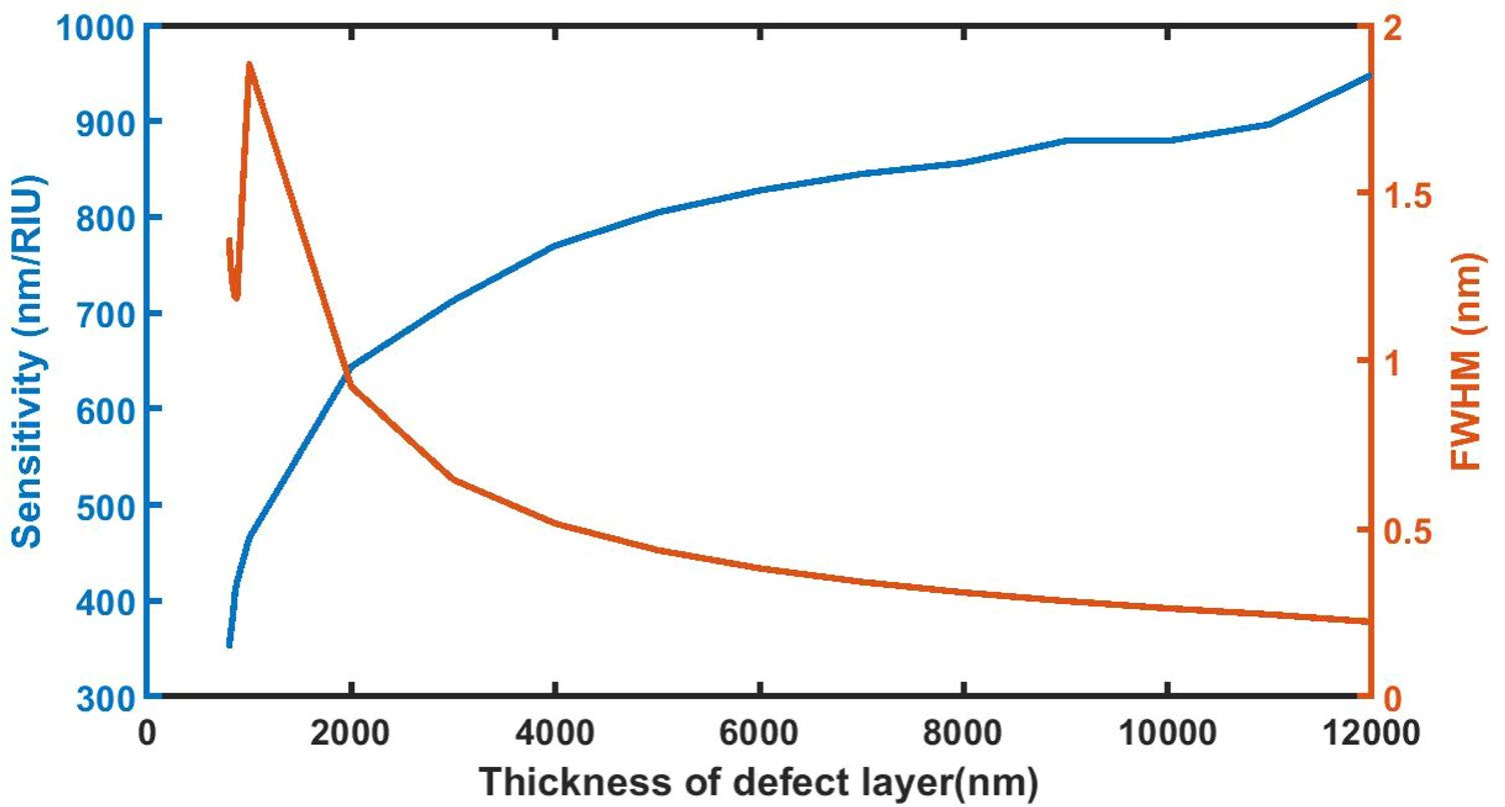
The dependence of sensitivity and full width half maximum of the defect layer on the thickness of defect layer region of 1d DPC (AB)^9^*D*(AB)^9^ loaded with the sample of refractive index 1.33087 under normal incidence.

Figure 7 depicts the dependence of the sensitivity and full width half maximum versus thickness of the defect layer region. Specifically, when the thickness of defect layer thickness increases from 800 to 12,000 nm, the sensitivity of sensor gradually increase from 350.6897 to 948.2759 nm/RIU while FWHM of defect modes associated with the sensor decreases from 1.3660 to 0.2225 nm. For defect layer thickness higher than 12,000 nm, the defect mode moves out of PBG and new defect mode appears into the PBG which have compromised values of both sensitivity and FWHM. The maximum sensitivity value of 948.2759 nm/RIU is achieved when the defect layer thickness of the sensor is set to *d*_D_ = 12,000 nm. So, the defect layer thickness of 12,000 nm is considered as an optimized thickness of structure. Under optimized cavity thickness the FWHM attains its minimum value of 0.2225 nm as required. Moreover this optimized defect layer thickness also improves the interaction between incident light and poliovirus sample due to the increase in the geometrical path difference between the light inside cavity which results significant enhancement in the sensitivity of the sensor.

### Effect of change in thickness of defect layer on *FoM* and *Q factor*

**Figure 8:**
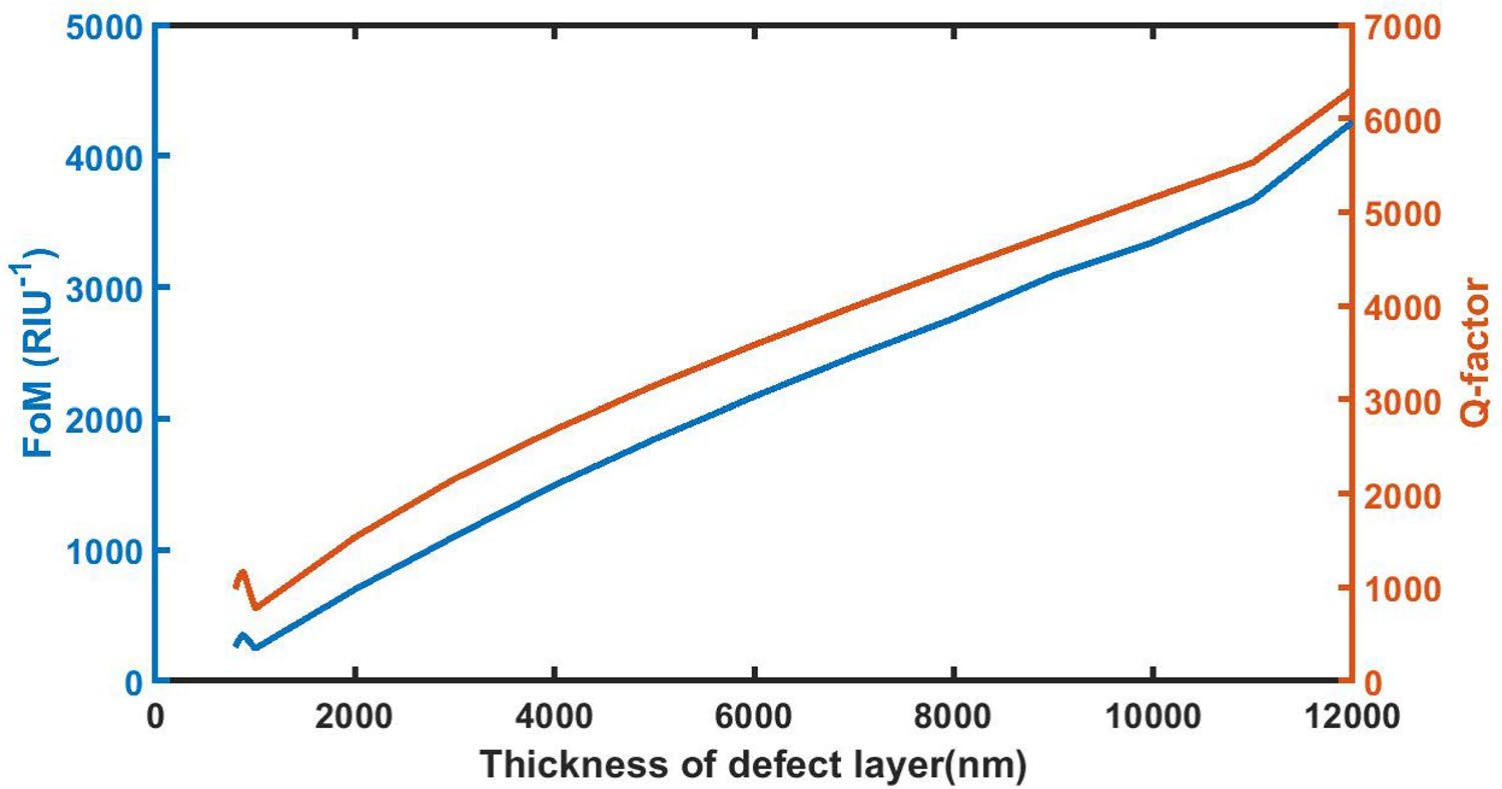
The dependence of figure of merit and quality factor on the thickness of defect layer region of 1d DPC (AB)^9^*D*(AB)^9^ loaded with the sample of refractive index 1.33087 under normal incidence.

Figure 8 shows the numeric values FoM and QF of the proposed 1d DPC (AB)^9^*D*(AB)^9^ loaded with sample of *C* = 0.005 g/ml at different defect layer thickness considerations from 800 to 12,000 nm. It appears from Fig. 7 that as *d*_D_ from 800 to 12,000 nm, FOM and QF values of the sensor gradually increases from 256.7312 per RIU to 4261.5154 per RIU and 979.5509–6312.2083 respectively. At *d*_D_ = 12,000 nm the FoM and QF values attain their maximum which indicates the best performance of the sensor.

### Effect of change in thickness of defect layer on *signal-to-noise* ratio and *dynamic range*

**Figure 9:**
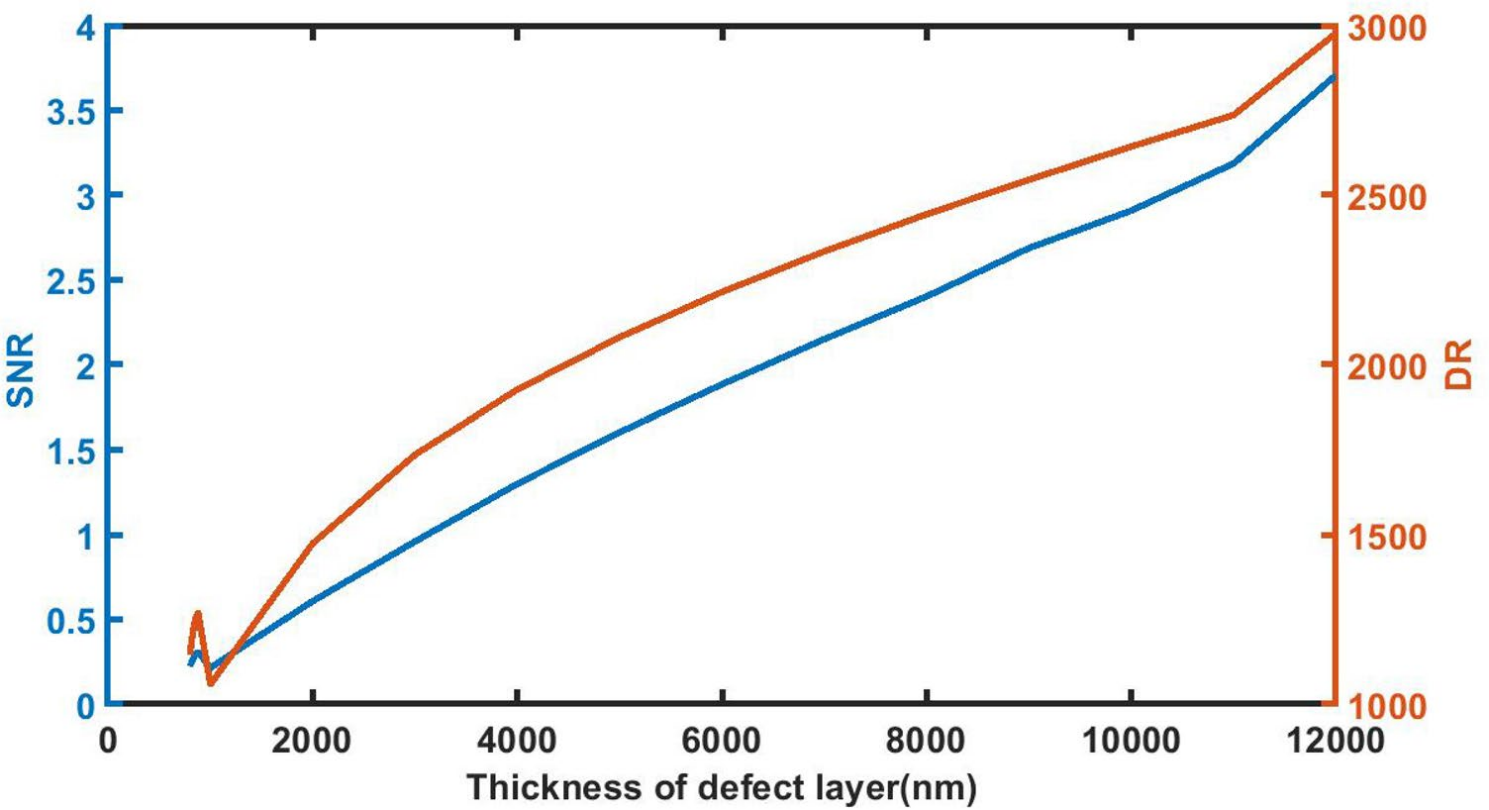
The dependence of signal-to-noise ratio and dynamic range on the thickness of defect layer region of 1d DPC (AB)^9^*D*(AB)^9^ loaded with the sample of refractive index 1.33087 under normal incidence.

Figure 9 indicates the numeric values of SNR and DR of the proposed 1d DPC (AB)^9^*D*(AB)^9^ loaded with sample of *C* = 0.005 g/ml at different defect layer thickness considerations from 800 to 12,000 nm. It can be seen from Fig. 8 that as *d*_D_ from 800 to 12,000 nm, SNR and DR values of the sensor gradually increases from 0.2234 to 3.7075 and 1144.8515 to 2977.6019 respectively. Thus with *d*_D_ = 12,000 nm the numeric values of SNR and DR attain their best which indicates the efficient performance of the sensor.

### Effect of change in thickness of defect layer on *limit of detection* ratio and *resolution*

**Figure 10:**
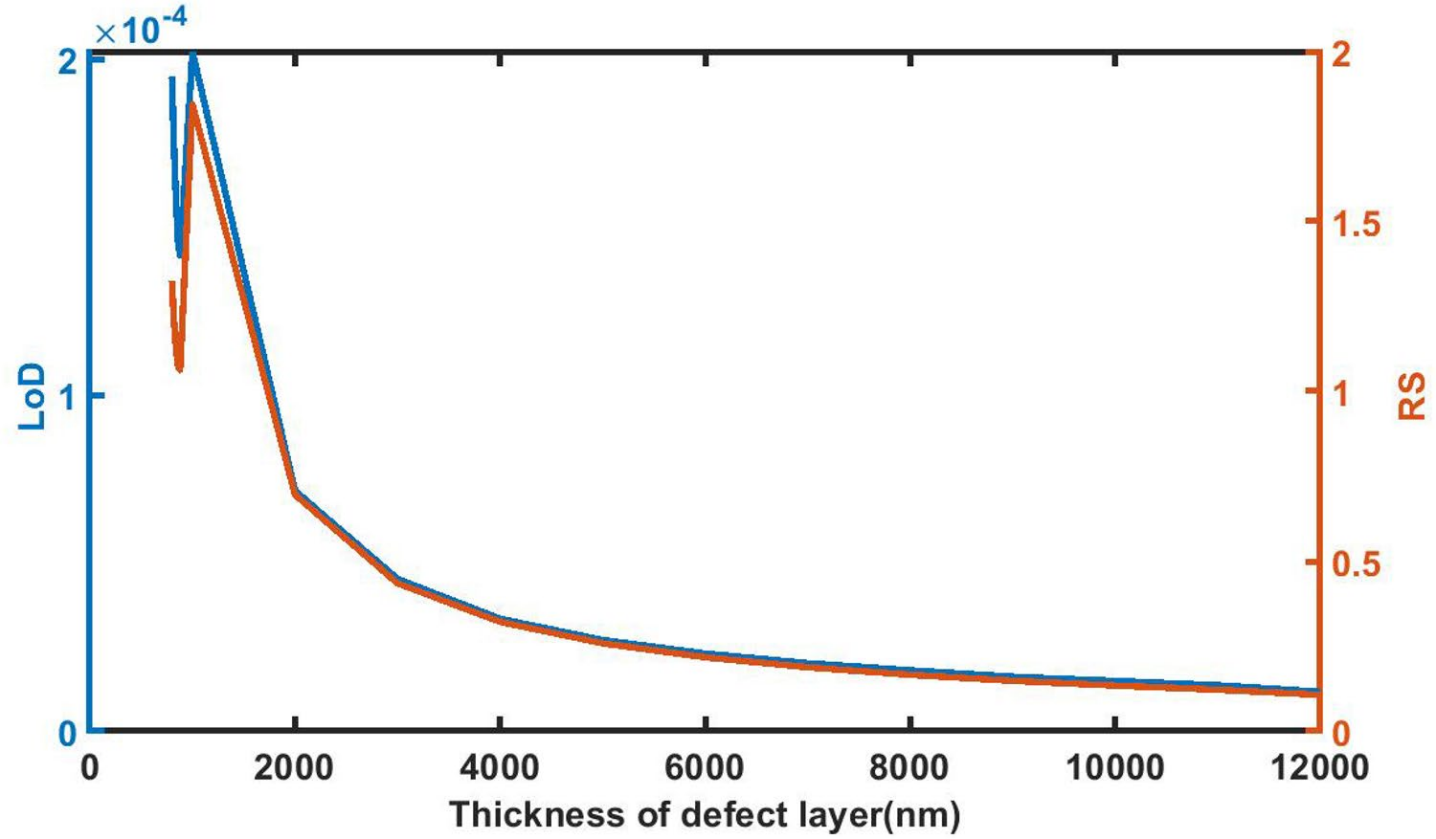
The dependence of limit of detection and resolution on the thickness of defect layer region of 1d DPC (AB)^9^*D*(AB)^9^ loaded with the sample of refractive index 1.33087 under normal incidence.

Figure 10 below shows the defect layer thickness dependent variations of LoD and RS of proposed 1d DPC (AB)^9^*D*(AB)^9^ loaded with sample of *C* = 0.005 g/ml. It shows that as *d*_D_ increases from 800 to 12,000 nm the LoD and RS values decline from 1.95E−04 to 1.17E−05 and 1.3247–0.1069 respectively. At higher thickness both values become constant which indicates that the further increase in the thickness of defect layer does not have any significant impact on LoD and RS values of the proposed sensing structure.

Thus the above analysis gives optimum value of defect layer thickness as d_D_ = 12,000 nm correspond to which the performance of the sensor is recorded as most heightened in terms of S, FWHM, FoM, QF, SNR, DR, LoD and RS.

### Optimization of period number *N*

After optimizing the thickness of the defect layer we have given our efforts to optimize the period number N of the proposed poliovirus sensor. For this purpose we have chosen the thickness of layers A, B and D of 1d DPC (*AB*)^N^*D*(*AB*)^N^ as *d*_A_ = 100 nm, *d*_B_ = 150 nm and *d*_D_ = 800 nm respectively. The sensor is loaded with analyte of refractive index 1.33087. The incident angle has been taken as θ = 0°. In order to obtain the optimized value of N we have calculated S, FWHM, FoM, QF, SNR, DR, LoD and RS values our design with the help of Eqs. (10)–(17). We have recorded the values of all performance evaluating parameters for each value of period number N which values from *N* = 5 to *N* = 10. These values are listed in Table 4 below.

**Table 4:** The effect of change in period number *N* of 1d DPC (AB)^N^*D*(AB)^N^ loaded with sample of *C* = 0.005 g/ml on the performance evaluating parameters of the proposed sensor under normal incidence with *d*_D_ = 12,000 nm.

N	FWHM (nm)	S (nm/RIU)	FOM (Per RIU)	QF	SNR	DR	LOD	RS
5	2.34732	954.0230	406.4308	598.8224	0.3536	917.4537	1.23E−04	2.0293
6	1.28472	954.0230	742.5939	1093.7255	0.6461	1239.6870	6.73E−05	0.9553
7	0.71184	948.2759	1332.1543	1973.5473	1.1590	1665.0896	3.75E−05	0.4574
8	0.39716	948.2759	2387.6241	3536.8044	2.0772	2228.9248	2.09E−05	0.2205
9	0.22252	948.2759	4261.5154	6312.2083	3.7075	2977.6019	1.17E−05	0.1069
10	0.12504	948.2759	7584.0000	11,233.1129	6.5981	3972.0829	6.59E−06	0.0520

### Effect of change in period number of the structure *N*on *S *and *FWHM*

**Figure 11:**
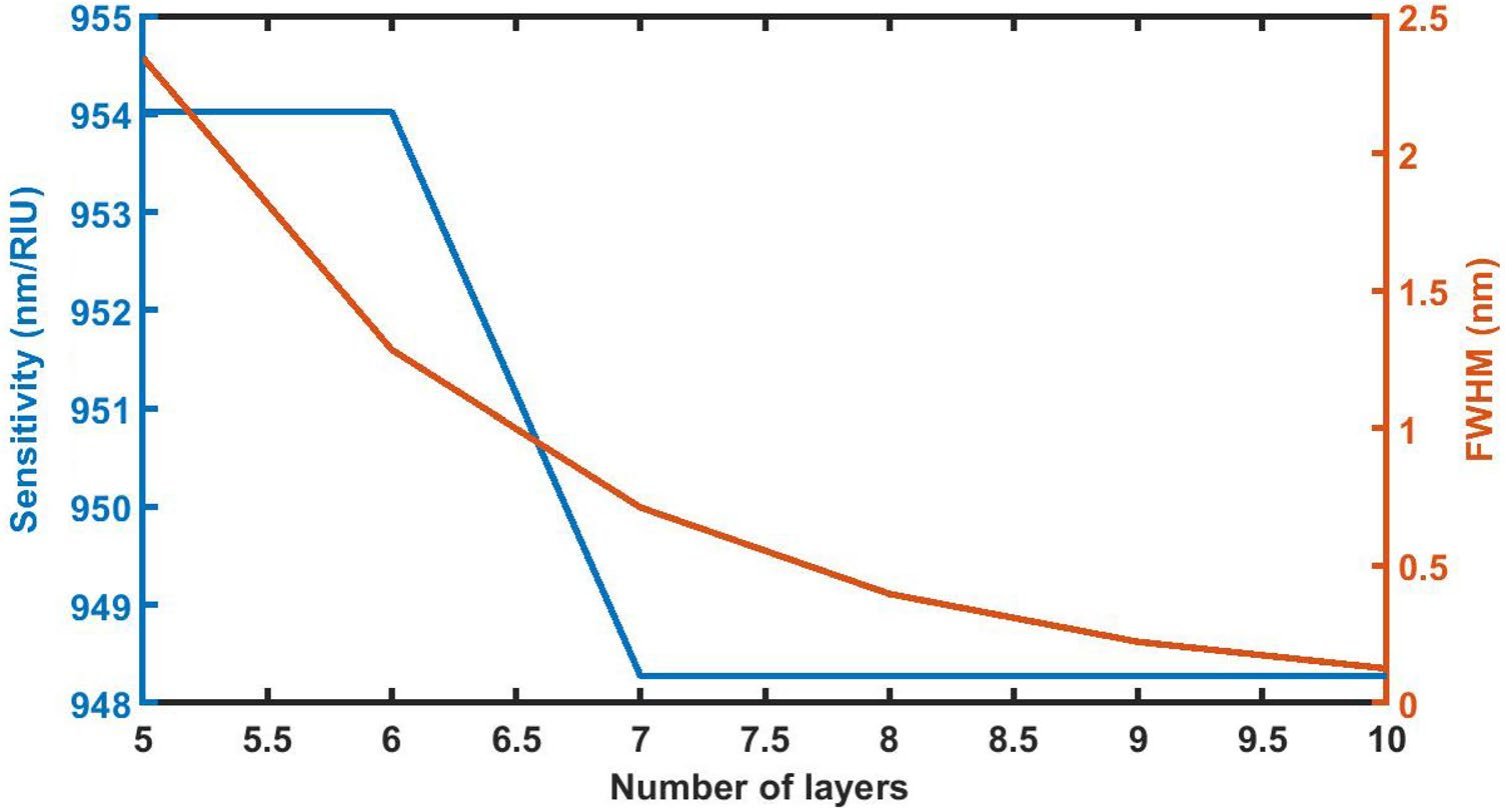
The dependence of sensitivity and full width half maximum of the defect mode on the period number *N* of 1d DPC (AB)^9^*D*(AB)^9^ loaded with the sample of refractive index 1.33087 under normal incidence with *d*_D_ = 12,000 nm.

Figure 11 demonstrates the change in the sensitivity and full width half maximum of defect mode of 1d DPC when the period number are *N* = 5, *N* = 6, *N* = 7, *N* = 8, *N* = 9 and *N* = 10. As the period number increases from *N* = 5 to *N* = 6, the sensitivity remains constant. It declines from 954.0230 to 948.2759 nm/RIU when *N* increases from 6 to 7. Further increase in *N* from 7 to 8, 9 and 10 does not have impact on sensitivity value corresponding to *N* = 7. The sensitivity value remains minimum of 948.2759 nm/RIU with *N* = 8, 9 and 10. As far as impact of period number on the FWHM is concern, it decreases from 2.34732 nm to 0.12504 nm with increase of period number. The decrease in the sensitivity of the structure is marginal as compared to the minimum value of FWHM corresponding to *N* = 10.

### Effect of change in period number of the structure *FoM* on *QF*

**Figure 12:**
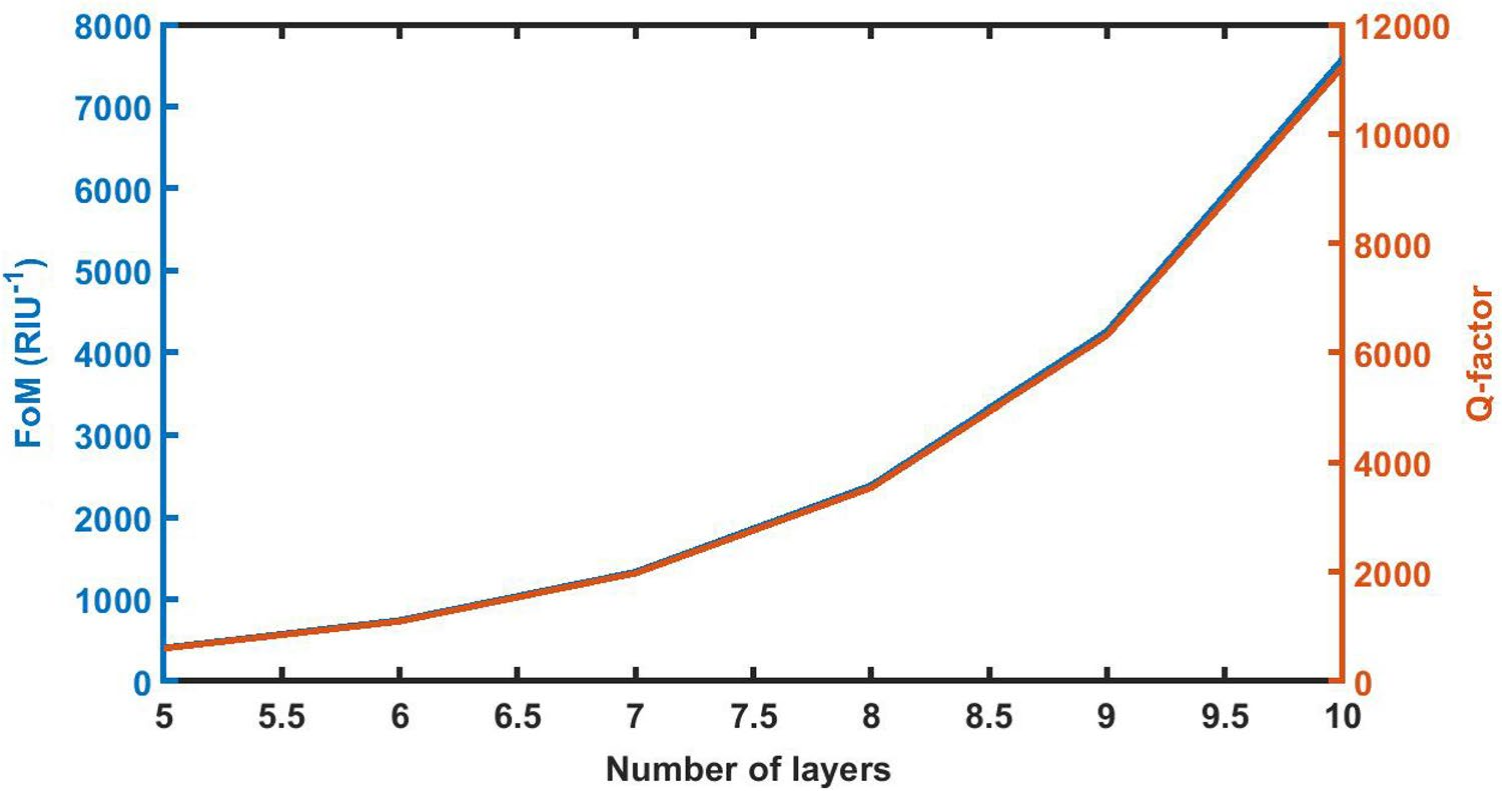
The dependence of FoM and QF on the period number *N* of 1d DPC (AB)^9^*D*(AB)^9^ loaded with the sample of refractive index 1.33087 under normal incidence with *d*_D_ = 12,000 nm.

The increase in period number of the structure from 5 to 10 in steps of 1 results the significant increase in the FoM and QF values of the proposed sensor as evident from Fig. 12. The maximum values of FoM and QF are always desirable for designing of high performance sensor. Our sensor reaches to maximum values of FoM and QF as 7584 per RIU and 11,233.1129 respectively with period number *N* = 10.

### Effect of change in period number of the structure *SNR* on *DR*

**Figure 13:**
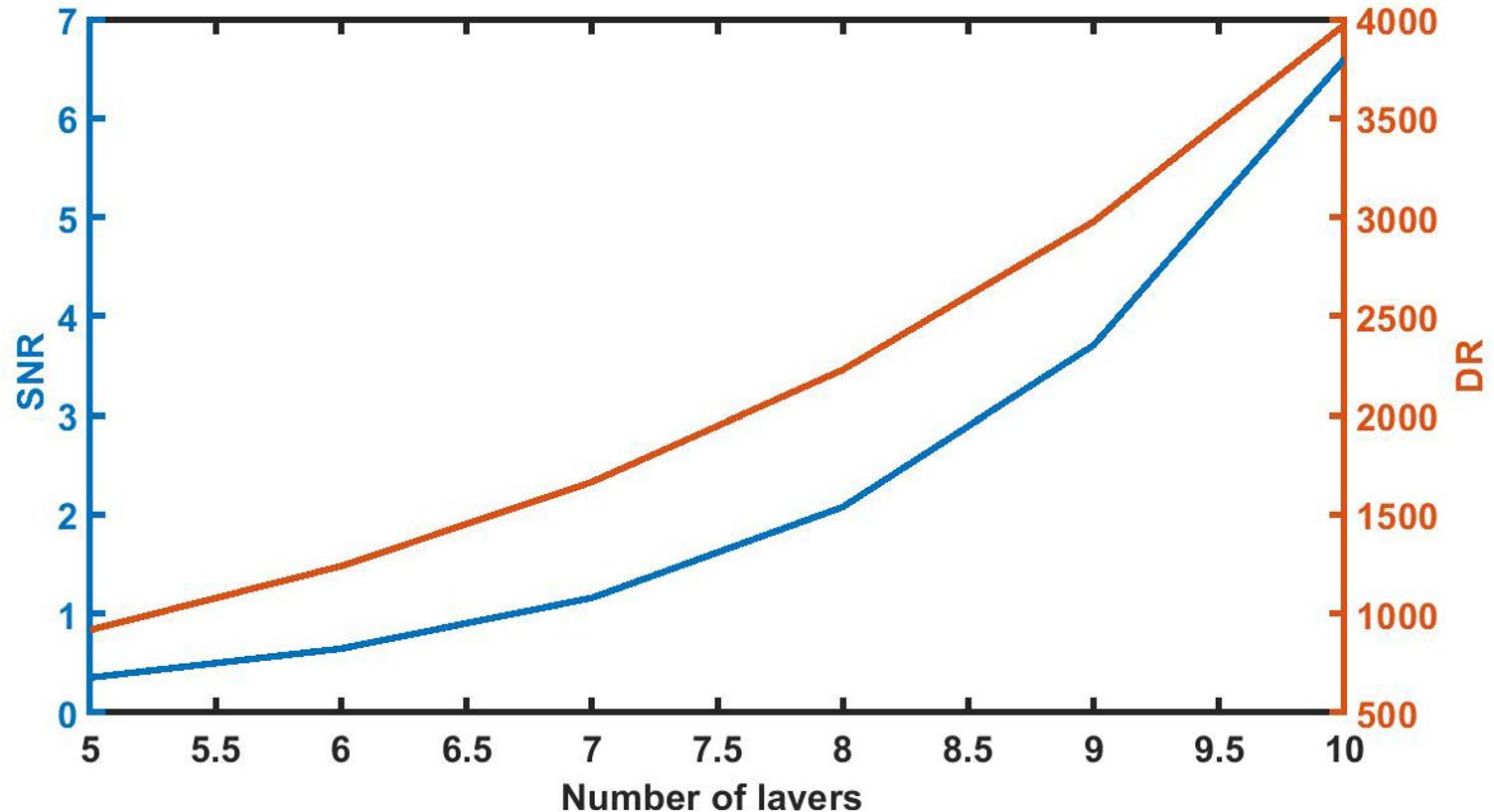
The dependence of SNR and DR on the period number *N* of 1d DPC (AB)^9^*D*(AB)^9^ loaded with the sample of refractive index 1.33087 under normal incidence with *d*_D_ = 12,000 nm.

Figure 13 indicates that as period number of the structure changes from N = 5 to N = 10, the signal-to-noise ratio and dynamic range starts to increase. Both the values of SNR and DR become maximum of 6.5981 and 3972.0829 respectively with *N* = 10.

### Effect of change in period number of the structure *LoD* on *RS*

**Figure 14:**
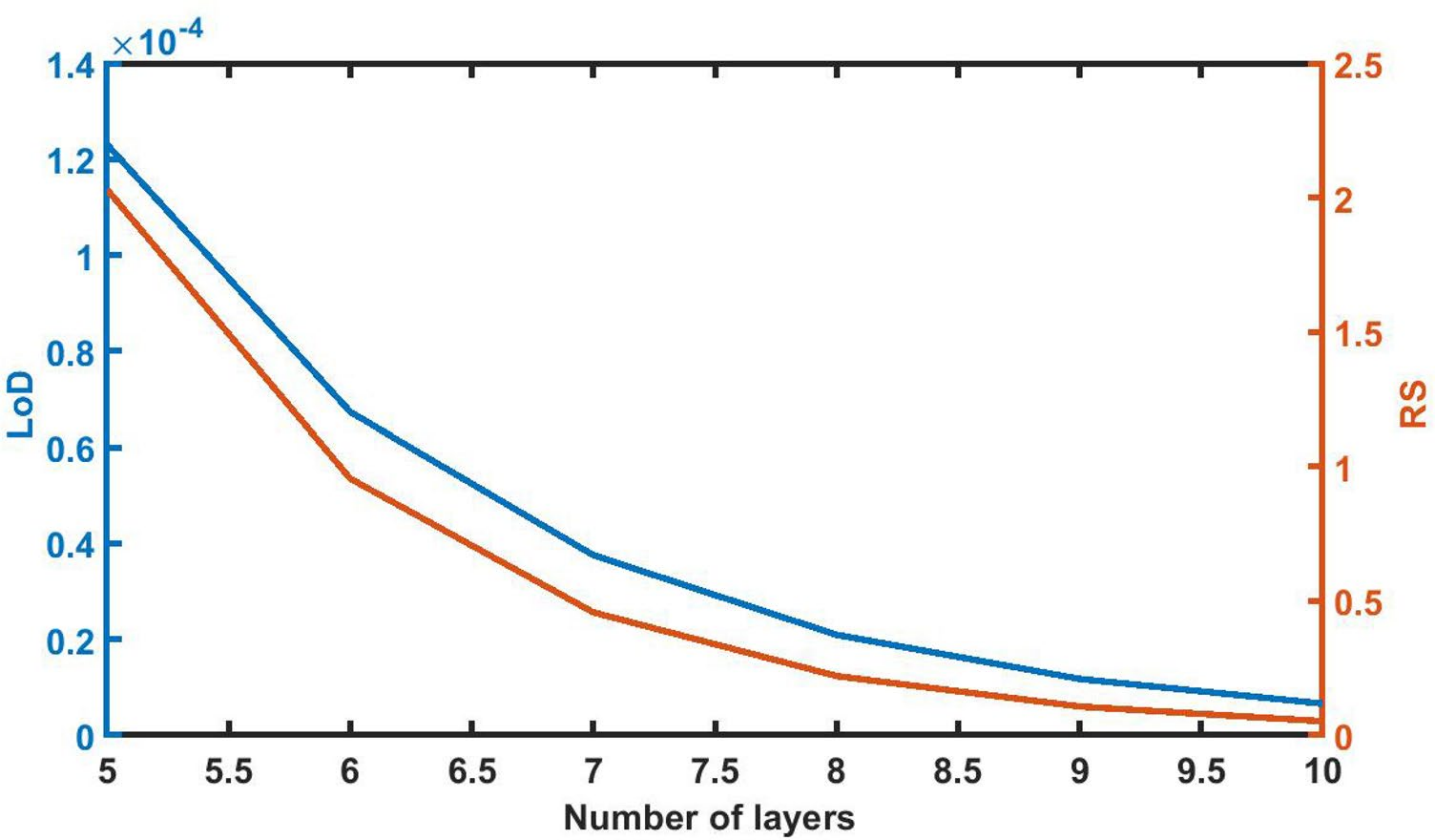
The dependence of LoD and RS on the period number *N* of 1d DPC (AB)^9^*D*(AB)^9^ loaded with the sample of refractive index 1.33087 under normal incidence with *d*_D_ = 12,000 nm.

The LoD and RS values of the poliovirus sensor gradually decreases as period number increases from N = 5 to N = 10 as evident from Fig. 14. The values of LoD and RS reach to minimum of 6.59E−06 and 0.0520 respectively with N = 10 as expected.

Thus with the help of studying the period number *N* dependent performance evaluation parameters *S*, *FWHM*, *FoM*, *QF*, *SNR*, *DR*, *LoD* and *RS* we have identified *N* = 10 as an optimum value of period number of proposed poliovirus sensor which corresponds to significant improvement in the performance evaluation parameters *FWHM*, FoM, QF, SNR, DR, LoD and RS except *S* of our design.

### Optimization of angle of incidence

After obtaining optimized values of defect layer thickness *d*_D_ and period number *N* of the proposed poliovirus sensor as a 12,000 nm and 10 respectively, we have extended our efforts to further improve the performance of the proposed design by changing the incident angle from *θ* = 0° to *θ* = 40°corresponding to TE wave. The change in the incident angle is one of an effective way of controlling and enhancing the performance of any sensor due to increase in the path length of the incident light into the sensor. Additionally, the increase in the incident angle also increases the confinement of light inside analyte present in the defect layer region of the structure. For the fulfillment of this goal we have change the incident angle from *θ* = 0° to *θ* = 40° corresponding to TE wave and study its effect on the performance evaluation parameters defined in Eqs. (10)–(17) above. The numeric values *S*, *FWHM*, FoM, QF, SNR, DR, LoD and RS of 1d DPC (AB)^9^*D*(AB)^9^ loaded with the sample of refractive index 1.33087 with *d*_D_ = 12,000 nm and *N* = 10 are summarized in Table 5 below. The variation in incident angle affects the defect mode position inside PBG of the structure.

**Table 5:** The effect of change in incident angle *θ* corresponding to TE wave of 1d DPC (AB)^*N*^*D*(AB)^*N*^ loaded with sample of *C* = 0.005 g/ml on the performance evaluating parameters of the proposed sensor with *d*_D_ = 12,000 nm.

Incident angle	FWHM (nm)	S (nm/RIU)	FOM (Per RIU)	Q	SNR	DR	LOD	RS
0°	0.12504	948.27586	7584.00003	11,233.11287	6.59808	3972.08294	6.59E−06	0.05201
10°	0.12473	954.02299	7648.53314	11,172.26143	6.65422	3945.76697	6.54E−06	0.05177
20°	0.18625	1017.24138	5461.57738	7606.42328	4.75157	3282.71706	9.15E−06	0.08410
30°	0.30511	1091.95402	3578.92287	4658.19669	3.11366	2573.02395	1.40E−05	0.15312
40°	0.45436	1189.65517	2618.28446	3102.06475	2.27791	2090.99500	1.91E−05	0.24656

### Effect of change in angle of incidence on *S *and *FWHM*

**Figure 15:**
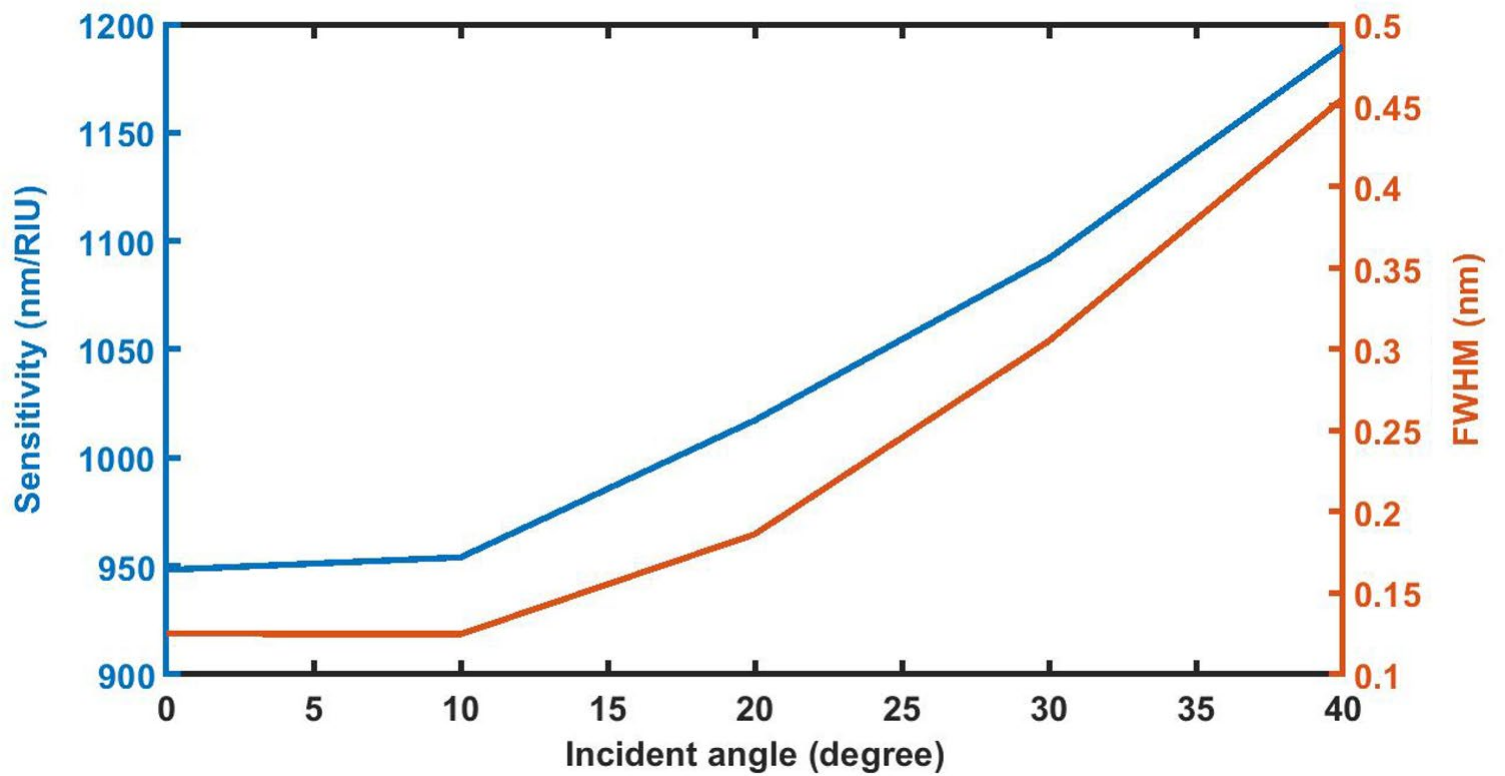
The dependence of sensitivity and full width half maximum of 1d DPC (AB)^9^*D*(AB)^9^ loaded with the sample of refractive index 1.33087 with *d*_D_ = 12,000 nm on the incident angle *θ* corresponding to TE wave.

Figure 15 below shows the variation of sensitivity and full width half maximum of 1d DPC (AB)^9^*D*(AB)^9^ loaded with the sample of refractive index 1.33087 with *d*_D_ = 12,000 nm on the incident angle *θ* corresponding to TE wave. It shows that the sensitivity and FWHM of the sensor increases with increase in the incident angle from *θ* = *0°* to *θ* = *40°*. For incident angles higher than *40°* defect mode disappears.

### Effect of change in angle of incidence on *FoM* on *QF*

**Figure 16:**
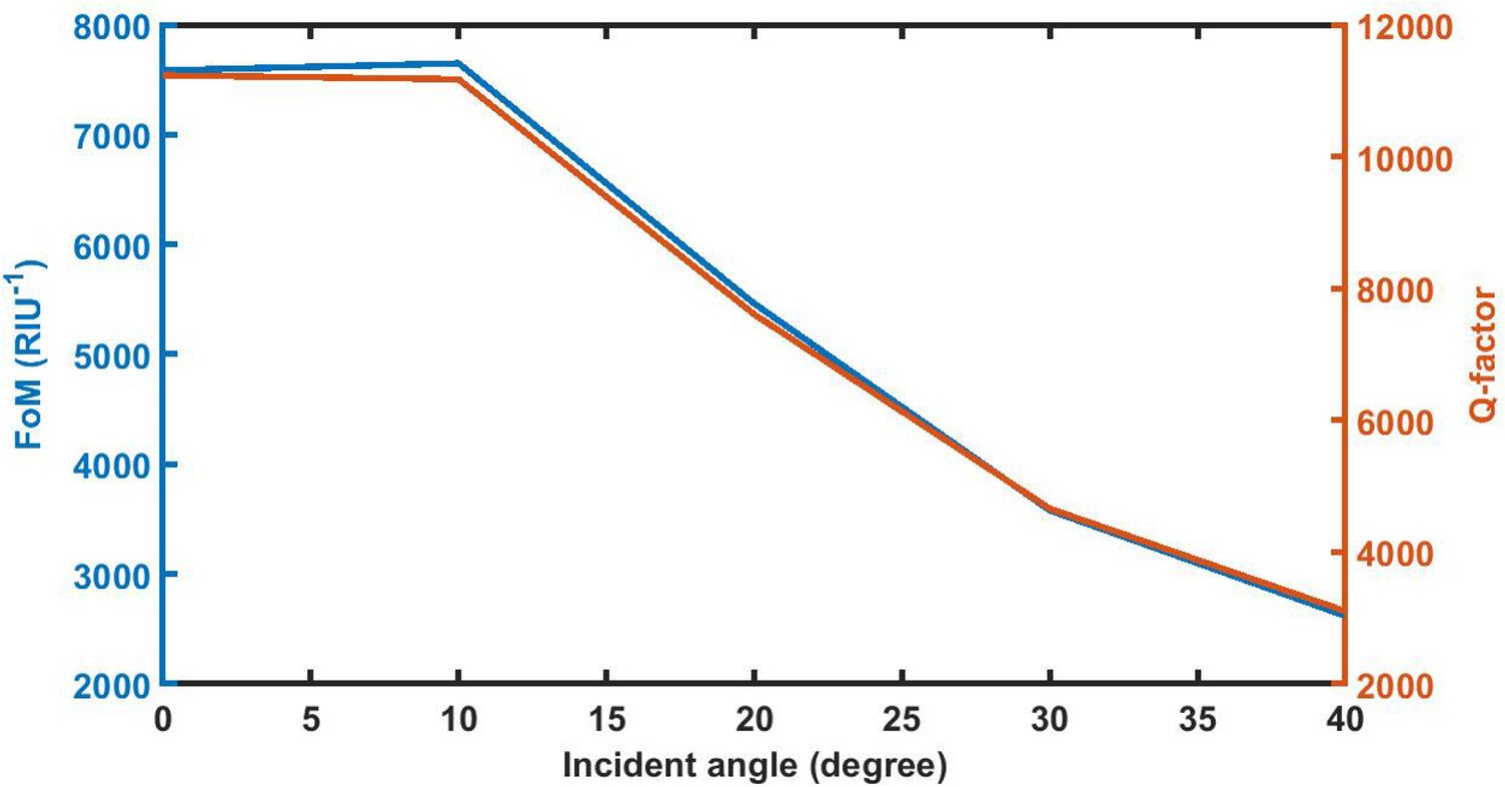
The dependence of FoM and quality factor of 1d DPC (AB)^9^*D*(AB)^9^ loaded with the sample of refractive index 1.33087 with *d*_D_ = 12,000 nm on the incident angle *θ* corresponding to TE wave.

Figure 16 below shows that the FoM and QF values of the poliovirus sensor initially remain constant by changing the incident angle up to 12°. Further increase in the incident angle from 12° results sudden change in the FoM and QF values of the sensor loaded with the sample of refractive index 1.33087 with *d*_D_ = 12,000 nm. At *θ* = 40° the FoM and QF values are reached to minimum of 2618.28446 and 3102.06475 respectively.

### Effect of change in angle of incidence on *SNR* and *DR*

**Figure 17:**
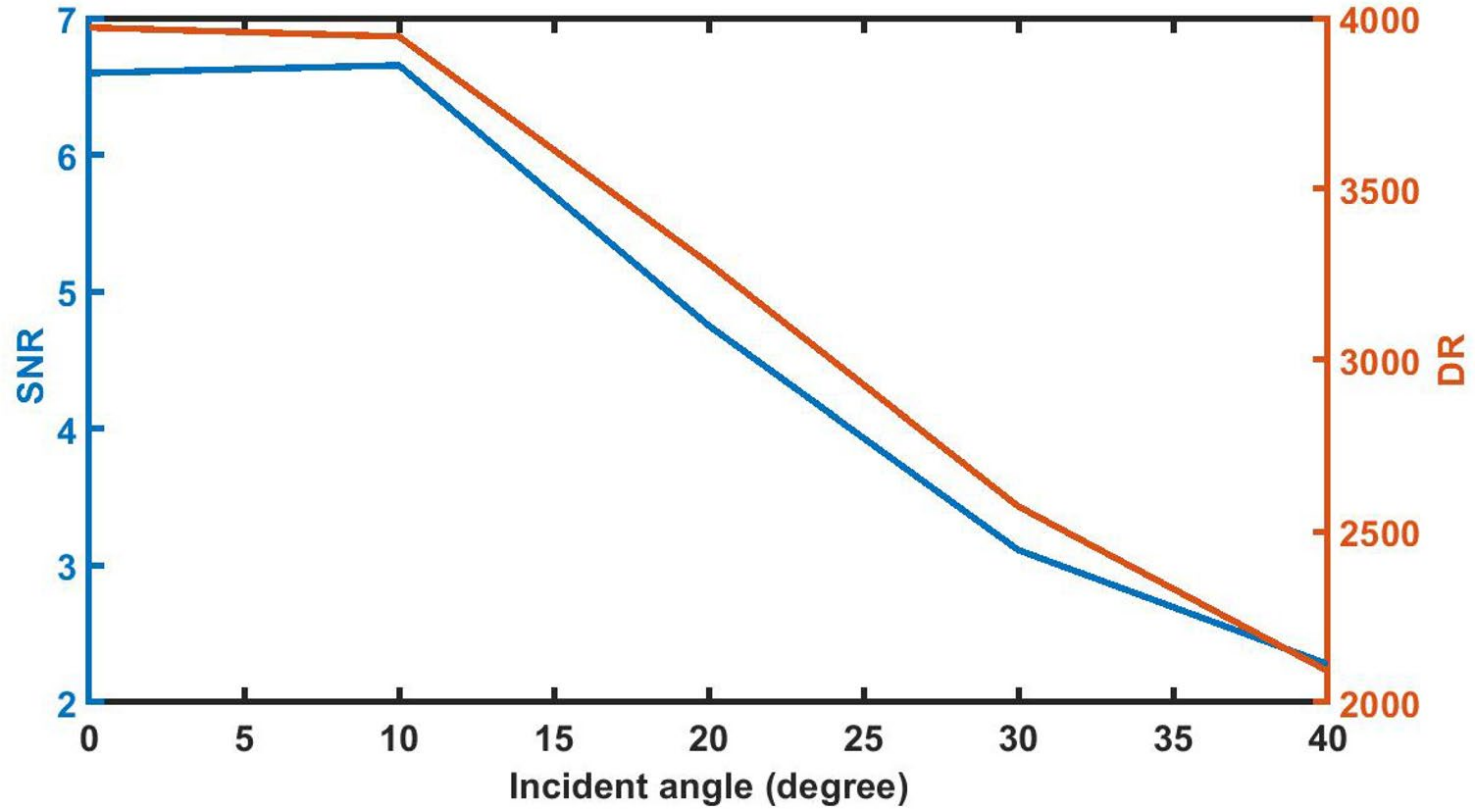
The dependence of SNR and DR of 1d DPC (AB)^9^*D*(AB)^9^ loaded with the sample of refractive index 1.33087 with *d*_D_ = 12,000 nm on the incident angle *θ* corresponding to TE wave.

Figure 17 below shows that the SNR and DR values of the poliovirus sensor initially have least variation by changing the incident angle up to 10°. Further increase in the incident angle from 10° results sudden change in the SNR and DR values of the sensor loaded with the sample of refractive index 1.33087 with *d*_D_ = 12,000 nm. At *θ* = 40° the SNR and DR values are reached to minimum of 2.27791 and 2090.99500 respectively.

### Effect of change in angle of incidence on *LoD* and *RS*

**Figure 18:**
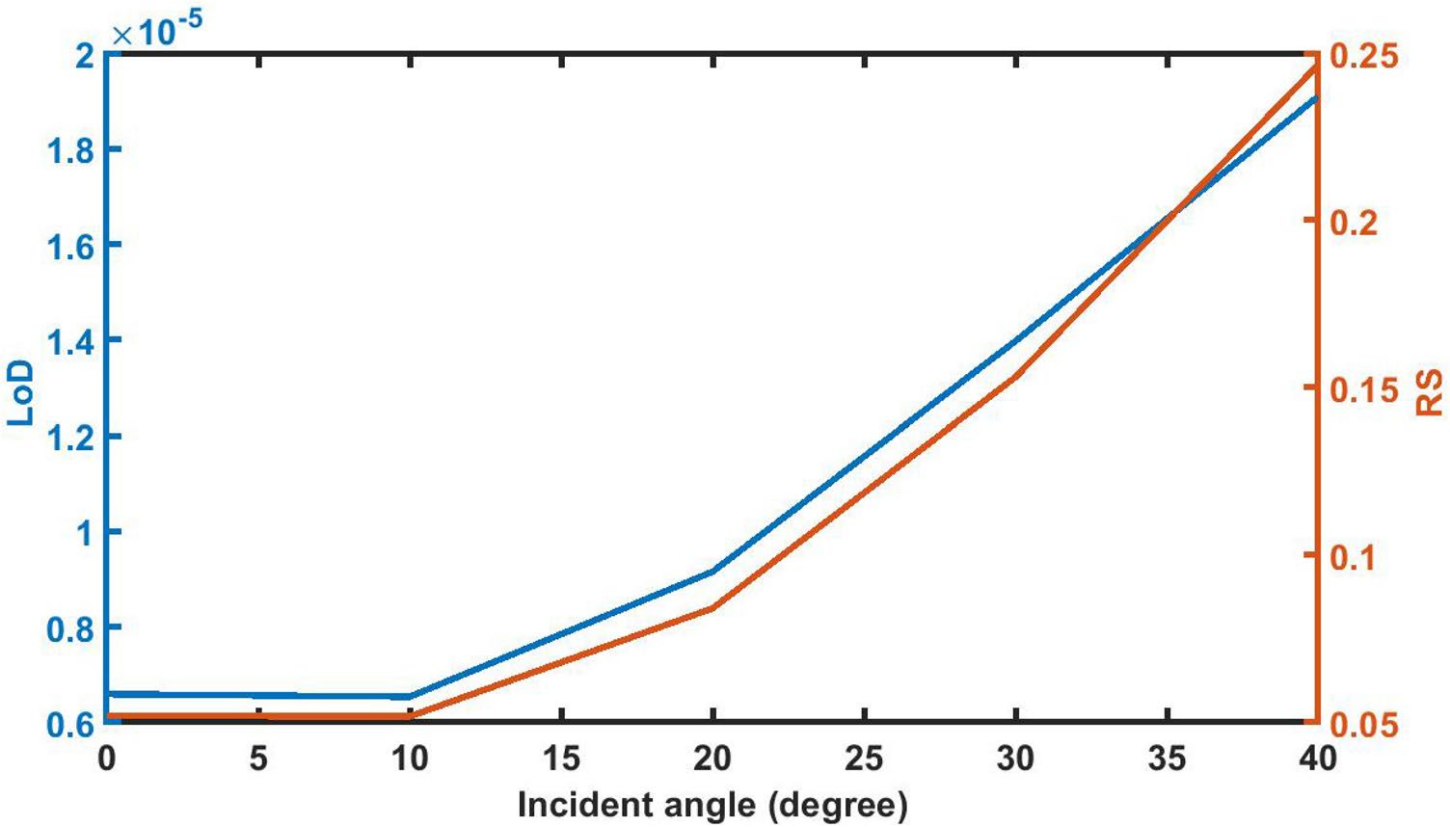
The dependence of LoD and RS of 1d DPC (AB)^9^*D*(AB)^9^ loaded with the sample of refractive index 1.33087 with *d*_D_ = 12,000 nm on the incident angle *θ* corresponding to TE wave.

Finally, the pictroial representation of LoD and RS dependent upon incident angle has shown in Fig. 18 below. It shows that any increase in the incident angle from 0° results signifcant increase in both these values. At θ = 40°, LoD and RS values become 1.91E−05 and 0.24656. These values are still small as desired.

Finally with the above analysis we have successfully optimized our sensor design for detection of poliovirus present in the water sample under consideration. The optimum values of defect layer thickness, period number and incident angle corresponding to TE wave are obtained as 1200 nm, 10 and 40° respectively. The selection of these optimum parameters associated with our structure makes the structure highly sensitive. The numeric value of sensitivity becomes 1189.65517 nm/RIU when the structure is loaded with sample of concentration *C* = 0.005 g/ml.

For providing better picture of the proposed work we have compared the proposed work with the work reported by other in recent years. Table 6 summarizes the comparison of performance between the structures proposed by us and other researchers in present years in terms of publication year of work, structural topology, sensitivity, figure of merit and quality factor. As evident from structures reported in references^39–41^ all have PbG topology, structures reported in reference 42 is based on plasmonics toplogy as well. The structures based on photonic, plasmonics and combination of both topologies have their advantages and disadvantages pertaining to the biosensing applications^41^. The transmission properties of the works presented in Table 6 are based on PbG. The structures whose transmission properties are based on PbG are good candidates for biosensing applications due to two main reasons. First, the edges of the PbG are sharp and second due to wider band gap for accommodating large number of analytes. The overlapping between high and low PbG due to change in the refractive index of analyte is one of major issue which must be addressed before using pc as a biosensor. For this reason the proposed work is being carried out in the near infrared region of the spectrum which is most suitable for biosensing applications. Due to the consideration of all above key points *S*, *FoM* and *QF* values of proposed photonic biosensor are exceptionally good and make our biosensor most sensitive and efficient as evident from comparison Table 6.

**Table 6:** Comparison between the performances of present and earlier reported sensing works based on sensitivity, figure of merit and quality factor.

Structure	Topology	Analyte refractive index range	*S* (nm/RIU)	*FoM* (Per RIU)	*QF*	References	Year
SOI based 1d pc	PbG	Sucrose (1.345–1.442)	1016.35	N.C	N.C	^39^	2021
2d pc	PbG	Red blood cells (1.336–1.399)	898.9764	N.C	212.771	^40^	2022
Biosensor I	PbG and plasmonics	Basal cell cancer (1.36–1.38)	718.6	156.217	207.4	^41^	2022
Biosensor II	PbG and plasmonics	Basal cell cancer (1.36–1.38)	714.3	60.1	80.16	^41^	2022
**Our work (1d PhC)**	**PbG**	**Poliovirus (1.33–1.338700)**	**1189.65517**	**26,180.23446**	**3102.06475**	**–**	**–**

*N.C.* not calculated

Significant values are given in bold.

## Conclusions

In this paper, a poliovirus sensor has been proposed for detection of virus concentration present in the sample by means of based on 1d dppc. The transmission spectra of this study have been obtained by using MATLAB software in addition to TMM. The contaminated water containing poliovirus sample is poured into the defect layer region of 1d dpc which shifts the location of defect mode from one position to another position inside PbG due to variation in the refractive index of water sample dependent upon the concentration levels of poliovirus present in the sample. The proposed sensing structure has been optimized to strengthen the sensing capabilities of the sensor by considering the effect of change in transmission spectra due to change in (1) defect layer thickness, (2) period number of the structure and (3) incident angle corresponding to TE wave. The adoption of these three approaches may bring the maximum sensitivity of 1189.65517 nm/RIU from the structure when the sensor is loaded with sample of concentration 0.005 g/ml. Though for achieving maximum sensitivity we have to tolerate with other performance evaluating parameters of the sensor, this compromise is not significant in comparison to the enhancement obtained in sensitivity.

## Data Availability

The data that support the findings of this study are available from the corresponding author upon reason-able request.
